# Singing Ability Assessment: Development and validation of a singing test based on item response theory and a general open-source software environment for singing data

**DOI:** 10.3758/s13428-023-02188-0

**Published:** 2023-09-06

**Authors:** Sebastian Silas, Daniel Müllensiefen, Reinhard Kopiez

**Affiliations:** 1 Goldsmiths University of London, London, UK; 2 Hanover Music Lab, Hanover University of Music, Drama and Media, Neues Haus 1, 30175 Hannover, Germany

**Keywords:** Singing test, Melodic memory, Similarity measurement, Music assessment, Melodic recall, Music psychology

## Abstract

We describe the development of the Singing Ability Assessment (SAA) open-source test environment. The SAA captures and scores different aspects of human singing ability and melodic memory in the context of item response theory. Taking perspectives from both melodic recall and singing accuracy literature, we present results from two online experiments (*N* = 247; *N* = 910). On-the-fly audio transcription is produced via a probabilistic algorithm and scored via latent variable approaches. Measures of the ability to sing long notes indicate a three-dimensional principal components analysis solution representing pitch accuracy, pitch volatility and changes in pitch stability (proportion variance explained: 35%; 33%; 32%). For melody singing, a mixed-effects model uses features of melodic structure (e.g., tonality, melody length) to predict overall sung melodic recall performance via a composite score [R^2^c = .42; R^2^m = .16]. Additionally, two separate mixed-effects models were constructed to explain performance in singing back melodies in a rhythmic [R^2^c = .42; R^2^m = .13] and an arhythmic [R^2^c = .38; R^2^m = .11] condition. Results showed that the yielded SAA melodic scores are significantly associated with previously described measures of singing accuracy, the long note singing accuracy measures, demographic variables, and features of participants’ hardware setup. Consequently, we release five R packages which facilitate deploying melodic stimuli online and in laboratory contexts, constructing audio production tests, transcribing audio in the R environment, and deploying the test elements and their supporting models. These are published as open-source, easy to access, and flexible to adapt.

It is almost too obvious to state: music must be produced to be perceived. Why then, have musical production tests, which capture enacted musical behaviors, been relatively underutilized compared to other forms of musical ability tests, which tend to focus on listening? Not only do perceptual musical ability tests disregard the participatory nature of music as an embodied activity (Leman & Maes, 2014), they may also discard useful information about the internal representations of a research participant (Silas & Müllensiefen, 2023). In this way, it has recently been emphasized that understanding the production of music is crucial to understanding musical ability in general (Okada & Slevc, 2021; Silas & Müllensiefen, 2023).

The general answer to the question we have posited is methodological: as outlined below, assessing produced musical behavior in a meaningful way is a difficult problem. The purpose of the present paper is to document the development of an archetypal form of music production test: a singing test. In doing so, we utilize it to better understand musical ability and, in turn, provide useful tools for music education. In order to do this, we bring together different perspectives and computational tools developed over the last few decades into one open-source, accessible framework. This should make music production research easier to conduct, and, consequently, help to understand musical abilities more comprehensively than before.

## Background

The main reason for the relative lack of studies of music production has been due to methodological limitations. Tests of music production are typically more difficult to implement and utilize for meaningful assessments of musical behavior, mainly due to the problem of so-called “dirty (or messy) data” (Müllensiefen & Wiggins, 2011; Silas & Müllensiefen, 2023; Sloboda & Parker, 1985). The primary issue with such data is obtaining useful symbolic representations of sound and music from recorded audio files. However, thanks to advances in technology (e.g., Ras et al., 2010), and the rise of open-source software in general, it is now possible to measure produced musical behavior in an objective and automated way, much more easily than before, and only contemporarily, across the Internet.

Since much of the general population do not play musical instruments on a regular basis (ABRSM, 2022), but almost everyone has some degree of musical ability (Honing, 2019; Müllensiefen et al., 2014), one inclusive way of assessing musical abilities in the general population is through the recording of singing. Research which employs singing, recorded as audio, as the main unit of analysis, generally has two separate strands in the research literature. In melodic recall research (Müllensiefen & Wiggins, 2011; Ogawa et al., 1995; Oura & Hatano, 1988; Silas & Müllensiefen, 2023; Sloboda & Parker, 1985; Zielinska & Miklaszewski, 1992), singing is used as a test of memory for melodies and can help understand how such memory develops over time. Alternatively, singing accuracy research (Pfordresher et al., 2010; Pfordresher et al., 2015; Russo et al., 2020; Tan et al., 2021) is generally concerned with the ability to sing accurately and how such knowledge can help improve singing education.

However, research into melodic memory or singing accuracy is usually conducted in isolation, without much interchange of knowledge between these two research areas. Yet, this is curious considering that the two domains are fundamentally connected. It may not be immediately obvious, but, like many other issues in music psychology (Silas et al., 2022), the causal relationships between singing ability and melodic memory can be argued in both opposing directions, depending on the perspective taken: a) lower-level singing abilities are required to sing pitches in tune, and hence, accurately demonstrate the melodic object held in memory (i.e., better singing produces a better melodic recall score) vs. b) in order to sing a melody well (or at all), one must be able to hold it in memory (i.e., better melodic memory produces better singing). Whilst b) is a plausible explanation from a cognitive perspective, from a data-driven and descriptive perspective, a) is important too, especially in the context of studying melodic memory, insofar as the ability of a participant to demonstrate their melodic memory is contingent on the accuracy of their singing. Consequently, we contend that, in order to understand melodic memory and/or singing accuracy well, both perspectives must be taken into account simultaneously. Furthermore, this should be the case with regards to both a testing framework and a supporting statistical modeling framework. We now briefly review the relatively disparate literatures in singing accuracy and melodic recall.

### Singing accuracy

The singing accuracy literature (e.g., Cohen, 2015; Pfordresher et al., 2010; Russo et al., 2020) is generally concerned with an individual’s ability to sing and what constitutes “good” singing. There have been several notable singing tests presented in the literature. The most well-known procedure is probably the *Seattle Singing Accuracy Protocol* (*SSAP*; Pfordresher et al. 2015) which is “intended to be both brief and highly specific in its focus” with respect to singing measurement (Pfordresher & Demorest, 2020). It is designed to measure how accurately one can reproduce musical pitches through singing, in the context of pitch-matching and also singing songs from memory. The procedure consists of participants completing the following tasks: i) vocal warmup tests comprised of singing a familiar song as well as a comfortable pitch, which is used to estimate a comfortable range for the participant to sing in; ii) singing ten pitches in their vocal range in relation to a vocal example; iii) singing a further ten pitches in relation to a piano tone; iv) imitating six four-note melodies; and v) singing a well-known melody. The *SSAP* incorporates other perceptual tests and questionnaires as part of the battery too. However, it is not open source, and as far as we are aware, is unavailable.

Similarly, in the *Sung Performance Battery* (Berkowska & Dalla Bella, 2013), first an assessment of participants’ vocal range is made, followed by five tasks: (i) single-pitch matching, (ii) pitch-interval matching, (iii) novel-melody matching, (iv) singing from memory of familiar melodies (with lyrics and on a syllable), and (v) singing of familiar melodies (with lyrics and on a syllable) at a slow tempo indicated by a metronome. Likewise, the *AIRS Test Battery of Singing Skills* (*ATBSS*) (Cohen, 2015; Cohen et al., 2020) measures the following abilities, to: i) sing internationally familiar songs (Brother John, Frère Jacques) as well as learn a new song; ii) perform short melodic fragments; iii) sing lowest and highest notes; iv) improvise the ending of a song, and v) create an entirely new song. In addition, several verbal recall tasks are included^1^.

More recently, and particularly relevant to our research, Tan et al. (2021) assessed a singing task’s validity and reliability in an online setting. This procedure included: i) singing *Happy Birthday*; ii) matching five notes and iii) singing unfamiliar seven-note melodies as well as including additional perceptual tests and questionnaires. Very recently, and innovatively, large-scale singing research has been also conducted online outside of the context of Western music (Anglada-Tort et al., 2022). Anglada-Tort et al. (2022)’s approach also uses automated scoring and an online testing environment, with the main task being to sing back short melodies as immediate recalls.

There is much overlap between the task procedures described above: single-note singing, familiar song singing, and melodic singing. The tasks we describe here share some similarities (e.g., single-note singing, melodic items), but have some differences. For instance, while possible in our framework, we do not ask participants to sing a familiar song in our default procedures. We also use a substantially larger and much more heterogeneous database of melodic items, from which we randomly sample, within constraints (e.g., a given melody length). These choices to maximize item feature variance reflect our interest in connecting melodic features to task performance via item response theory (De Boeck et al., 2016).

An important point to note is the fact that singing accuracy research is more concerned with fine-grained pitch control compared to melodic memory research, which is about understanding high-level melodic mental representations. However, even singing accuracy appears to comprise two slightly disparate skills: accuracy (proximity to a target) and precision (consistency of reproduction) (Pfordresher et al., 2010). This highlights the need to not only measure singing accuracy and melodic memory via sung recall simultaneously but also several constructs related to singing accuracy simultaneously.

### Melodic recall

In contrast to singing accuracy tests, the *melodic recall paradigm* was designed as a test of melodic memory, with the most cited early example being Sloboda and Parker (1985). The melodic recall paradigm is used to make inferences about melodic memory, its errors, and how melodic representations build up over time (Müllensiefen & Wiggins, 2011; Silas & Müllensiefen, 2023). It does not usually include specific metrics related to singing accuracy. There have been several studies using this paradigm as a melodic memory test (e.g., Silas and Müllensiefen, 2023; Ogawa et al., 1995; Oura and Hatano, 1988; Zielinska and Miklaszewski, 1992) and several insights can be drawn from this research: 1) when learning a melody, harmony may be extracted more readily than rhythm or interval information (at least for participants with a substantial amount of prior musical training); 2) over successive attempts, participants store more notes in memory and try to recall more on each attempt; 3) participants generally get better at singing melodies over multiple attempts (Silas & Müllensiefen, 2023).

### “Dirty” musical data and similarity assessment

Despite the importance of produced actions in musical behavior, there is a relative dearth of research investigating musical recall and production compared to studies using purely perceptual paradigms to investigate melodic processing (e.g., Idson and Massaro 1978; Dowling and Fujitani 1971). One proposed reason for the scarcity of melodic recall studies is that melodic production data is relatively “dirty” (i.e., not easy to clean and analyze) and difficult to model (Müllensiefen & Wiggins, 2011) since it requires the transcription of a recorded signal to a symbolic representation (e.g., musical notation or numerical representations) from audio files. However, thankfully, in recent years, much progress has been made in this domain (Kim et al., 2018; Mauch & Dixon, 2014).

As originally conveyed by Sloboda and Parker (1985), and more recently articulated by Müllensiefen and Wiggins (2011), so-called “dirty” data usually requires “expert interpretation. Participants are required to sing, and their singing may be inaccurate; in some places, it is necessary to infer which note(s) they meant to sing. The participants’ singing is recorded, and it is possible that the recording may be imperfect”. Such imperfect singing is also surely related to the amount of effort expended by a participant, a perennial issue for performance research in general (Silm et al., 2020). The issue of effort and motivation affecting performance outcomes is very difficult, or impossible, to entirely mitigate, especially in the context of online research.

Furthermore, issues beyond audio transcription arise, once symbolic representations have been created: how should somebody’s recall be assessed with respect to a target melody, especially when sung recalls may greatly differ in length from the target melody for comparison (Müllensiefen & Wiggins, 2011; Silas & Müllensiefen, 2023)? To approach this issue, Müllensiefen and Wiggins (2011) and Silas and Müllensiefen (2023) utilized a computational approach which quantified sung recall performance in terms of a melodic similarity metric, providing a more objective and transparent approach than the prior approach of using human judgements to assess the similarity between target melody and (imperfect) recalls, or utilizing accuracy measures (Sloboda & Parker, 1985), which are inadequate (Silas & Müllensiefen, 2023). Likewise, as articulated in detail in Experiment 2, we propose the *opti3* melodic similarity metric (Müllensiefen & Frieler, 2004a) as being a suitable metric for scoring melodic sung recall data.

In addition, our framework mitigates the impact of dirty data early in the stage of data collection through several features of the online test interface, such as real-time signal-to-noise (*SNR*) measurement of a participant’s environment, to triage participants with noisy rooms, as well as transposing stimuli into the computed singing range of a participant, to ensure that their ability is not underestimated by presenting out-of-range stimuli (see Table 11 for a list of technical and procedural features for reducing noise in the collection of singing data online).

### Integrating singing accuracy and melodic recall

To integrate singing accuracy and melodic recall perspectives, Pfordresher et al. (2015)’s cognitive model of singing accuracy provides a useful framework for understanding accurate singing and melodic production. At the low level, this model comprises an auditory feedback loop. In this loop, first, external auditory input is processed as low-level perceptual representations of sound (pitch, duration, timbre, loudness). Such low-level representations are used as input to a translation model, which relates auditory input to sensorimotor action that is relevant to singing. Hence, this enables the guidance of a singer’s sensorimotor plans to adjust their singing (e.g., to be in tune), in response to auditory feedback. Such changes in sensorimotor actions comprise physical processes like respiration, phonation, and articulation. The lower-level auditory representations are also used as input to higher levels of cognition, which hold mental templates about music (e.g., its features, such as its tonality), stored in long-term memory (Baddeley et al., 2009). These templates allow auditory content to be categorized, forming more sophisticated representations of it, taking on musical domains such as representations of (melodic) features like tonality and contour, as well as segmenting melodies into coherent perceptual chunks. These formed higher-level representations can in turn be used as input back to the lower-level auditory feedback loop and further inform sensorimotor planning. Hence, the overall architecture of Pfordresher et al. (2015)’s cognitive model is bidirectional: both “top down” and “bottom up”. Altogether, this system enables a singer to fulfill objectives related to sung recall (i.e., hearing stimuli, representing its musical features mentally, responding through singing, and adjusting behavior to fulfill the goal sufficiently).

Our focus in the current paper is on the higher-level aspects: memory for melodic representations. However, we also simultaneously seek to take lower-level singing accuracy into account. In this way, we aim to draw the literature from both research areas described above and integrate them comprehensively, as Pfordresher et al. (2015)’s model suggests. To meet this objective formally, we invoke item response theory (IRT; De Boeck et al., 2016) as a psychometric modeling framework.

### Cognitive modeling via item response theory

Performance on an ability test can vary as a function of individual differences (i.e., some participants have a higher ability than others), but also as a function of items themselves (i.e., some items may be more difficult than others). In our study, there are two broad trial types: single long note singing and melodic singing. If long notes are presented in the vocal range of a participant (as we do here), the “item” effect of long notes are not expected to be important. That is: certain single pitches do not have properties, which make them more or less difficult to sing than others.

Conversely, for melodic items with multiple notes, musical features emerge (e.g., tonality, contour, rhythm). Such emergent features clearly rely on high-level mental representations and templates (i.e., musical knowledge). Consequently, there can be significant variance in complexity when a melody is the item of testing, and these kinds of item difficulties are important to model. Important melodic representations can be quantified for each melodic item across important dimensions (Müllensiefen, 2009). As suggested by previous literature (Baker, 2019; Dreyfus et al., 2016; Harrison et al., 2016), there are several melodic features that could indicate an item’s complexity and predict singing performance (e.g., tonality, interval contour, a melody’s frequency in occurrence).

In order to formally relate structural features of melodies to the cognitive difficulty of melody processing, the main methodological approach we utilize here is explanatory item response theory (IRT; De Boeck et al. (2016)). In this paper, IRT can be considered our first level of modeling, where melodic features become predictors of the *opti3* similarity score, which we take as representing variance in both singing accuracy and melodic memory. IRT is useful for our enquiry since it allows the simultaneous modeling of item difficulties and individual differences together via mixed-effects modeling, while compartmentalizing the variance into fixed item effects (melodic features), random item effects (unexplained effects due to melodic items), and participant effects (effects due to individual participants’ abilities). Additionally, an IRT model can be the basis of creating an adaptive test, which is highly efficient and can be variable in test length, since encoding relationships between item features and performance can be used to generate or select items based on modeled difficulties (for similar approaches see Gelding et al., 2021; Harrison et al., 2017; Harrison & Müllensiefen, 2018; Harrison et al., 2016; Tsigeman et al., 2022). Such an adaptive test can hence be employed flexibly, with potential applications in education.

In this paper, our strategy to relate singing accuracy to melodic memory is to extract participant- and item-level scores from our IRT mixed-effects models and use these outputs in further modeling. For instance, we use participant-level scores to represent individual differences in overall melodic memory and singing ability, and participant-level indicators of singing accuracy alone (comprising e.g., single long note singing, singing accuracy, precision), to predict such outputs. This allows us to evaluate the potential extent that low-level singing abilities are responsible for the overall variance in singing performance, leaving the rest to do with variance in melodic memory, or being unexplained.

### Motivations

The research presented here offers a two-fold contribution to the research literature, in both terms of: (1) general accessibility and (2) methodological advances.

#### An accessible open-source framework

While developed literature already exists regarding melodic recall (Müllensiefen & Wiggins, 2011; Silas & Müllensiefen, 2023; Sloboda & Parker, 1985) and singing accuracy (Cohen, 2015; Pfordresher et al., 2010; Russo et al., 2020), and there are several previous singing tests described in the literature (e.g., Pfordresher & Demorest, 2020; Tan et al., 2021), we do not know of a transparent and flexible open-source framework that enables researchers to easily access and implement a singing test themselves, enabling laboratory and online data collection for several simple singing tasks, such as sustaining long notes and imitating melodies. The main contribution of the present research is simply to make such a tool accessible to a wider audience and remove the “black box” element that masks many described singing paradigms in the literature. This can be considered an important step towards the standardization of singing tests.

#### Methodological advances in sung recall research

In terms of more sophisticated usage, and advancing previous methodologies and theoretical insights, like other innovative recent research (Anglada-Tort et al., 2022; Jacoby et al., 2019), our test framework and approach also makes a number of important contributions beyond its accessibility. These are to: (a) enable the automatic filtering of participants based on background noise at the beginning of a test; (b) present stimuli based on the participant’s empirically determined vocal range; (c) support multiple languages^2^; (d) comprehensively integrate melodic recall and singing accuracy frameworks to assess both low-level singing ability and high-level melodic memory ability simultaneously; (e) allow easy implementation in a timeline together with other behavioral tests; (f) be readily usable with new item banks of melodic stimuli^3^; (g) be able to provide real-time feedback for possible extensions to educational settings; (h) be supported by statistical models which connect relevant melodic recall and singing accuracy variables to one another, at both the trial level and beyond, and to do so; (i) be based on item response theory (IRT; De Boeck et al. 2016) as a statistical modeling framework, which allows us to construct a computerized adaptive (Harrison, 2018) version of the test that can be extended to educational settings.

In turn, we hope that the framework’s accessibility and methodological advances will go hand-in-hand, and stimulate the solving of more complicated issues in sung recall research, such as improving the quality of sung audio transcription. Having an open-source infrastructure in place enables researchers to start from the principle of tackling such issues from the outset, without the large startup cost of implementing singing technology in the first place.

### The present study

Hence, the main objective of this research was to draw upon the literatures in melodic recall and singing accuracy and build a new, easily accessible computational ecosystem for conducting melodic recall and singing research simultaneously (or in isolation, if desired). We also provide detailed documentation on how to access, utilize, and adapt the software with reference to web documentation^4^. Moreover, in the spirit of open science, the framework is completely open source, and hence transparent; it additionally has the flexibility to be adapted (e.g., to score sung recall data with new custom measures) by being constructed in a modular way, allowing new research ideas to be taken forward more easily than before.

To that end, we designed a test, and complementary open-source testing environment of sung recall, which we called the *Singing Ability Assessment* (SAA). The protocol incorporate three sets of procedures and underlying statistical models which reflect (1) single long note singing ability, (2) rhythmic melodies singing ability, and (3) arhythmic melodies singing ability as separate trial blocks. These are realized in five key open-source *R* packages: (1) *pyin*^5^, which wraps the *pYIN* (Mauch & Dixon, 2014) and *Sonic Annotator* (Cannam et al., 2010) libraries in *R*, enabling fundamental frequency and note onset estimation computation within the *R* environment (using *pYIN*); 2) *musicassessr*^6^, a general environment for deploying musical stimuli and collecting musical data in *psychTestR* (Harrison, 2020), especially for scoring melodic production and singing data^7^; (3) *itembankr*^8^ for creating useful item banks of melodic stimuli for use with *musicassessr*/*psychTestR*; (4) *Berkowitz*^9^, an item bank of melodies from Berkowitz et al. (2017; see also Baker, 2021) as outputted by *itembankr*, that can be used in *musicassessr* tests and (5) the *Singing Ability Assessment* (SAA) test package^10^ which brings everything together and allows for the comprehensive, yet efficient, collection of singing data. We invite collaborators to explore and contribute to these open-source packages.

Across two experiments, we describe the development of the *Singing Ability Assessment* (SAA). Experiment 1 describes a preliminary “rehearsal” paradigm for testing melodic singing, validated alongside other measures of musical and non-musical abilities. Experiment 2 describes updates to the protocol which utilizes a new paradigm (the “one-shot” paradigm) and allow all scoring to be done on-the-fly. Moreover, we also validate the new singing test alongside other measures of singing accuracy described in the literature, and hence, formally associate singing accuracy and melodic recall variables together. Both experiments result in item response theory (IRT; De Boeck et al. 2016) models to support the paradigms.

### Experiment 1: Design, development of and calibration of the Singing Ability Assessment (SAA) task

In Experiment 1, we aimed to design a new test for capturing sung recall. We wanted the task to share a seamless integration with statistical modeling packages (such as those which implement item response theory approaches) as well as other musical ability tests. In this regard, the *psychTestR* (Harrison, 2018; Harrison, 2020) framework in the *Shiny* (Chang et al., 2019) and *R* (R Core Team, 2020) environments was ideal since many statistical tools and validated musical and non-musical ability tests are now implemented in this framework^11^.

After developing the new *SAA* task, Experiment 1 seeks to validate the SAA via the construction of an explanatory IRT model, which is a special case of a general linear mixed-effect model Boeck et al. (2011). As a means of determining construct validity, we then sought to correlate its derived ability score with other previously validated ability tests, described below.

#### Hypotheses

 Performance on the SAA was hypothesized to be predicted by structural features of the melodic stimuli, which indicate melodic complexity (e.g., tonality and interval contour; see Dreyfus et al. (2016), Fleurian et al. (2017) and Müllensiefen and Halpern (2014) for similar approaches).

Secondly, in addition to structural features of melodies, we hypothesized that performance on our new test would also be related to individual-difference scores on other questionnaires/tests of related musical and non-musical abilities, in line with Pfordresher et al. (2015)’s model and the other literature reviewed above. These were measures of: working memory capacity (Tsigeman et al., 2022), pitch discrimination (a *psychTestR* re-implementation of Soranzo & Grassi, 2014)^12^, mistuning perception (Larrouy-Maestri, Harrison, & Müllensiefen, 2019), melodic discrimination (Harrison et al., 2016) and pitch imagery (Gelding et al., 2021). Relationships of our derived SAA score with these other indicators would offer concurrent validity to the novel task.

### Method

#### Participants

A total of 247 participants aged 18–77 (*M* = 29.06, *SD* = 11.98; 60% female; 3 = “Other”; 1 = “Prefer not to say” and ten missing, reason unknown) were recruited through social media and the marketing panel *SliceThePie*^13^. A subset of 72 of these participants completed an extended procedure with several more tests (described below) than the main sample.

### Materials

#### Singing Ability Assessment (SAA)

We extended *psychTestR*’s capabilities by adding in-browser audio recording functionality and on-the-fly in-browser melody playback (“Tone.js,” 2020). The task was deployed on an *Amazon EC2*^14^ server instance which hosted a Shiny server^15^ environment. Participant response was recorded as audio in the Internet browser and sent to an *Amazon S3*^16^ media storage bucket, where it was later downloaded for analysis^17^. Utilizing browser feature detection (“Modernizr,” 2020), users whose browsers did not support the necessary features (e.g., the “MediaRecorder” browser feature) were not allowed to enter the test. Participants were given an opportunity to test their microphone and headphones. They selected a vocal range that best matched their voice with reference to audio examples (*Soprano*, *Alto*, *Tenor*, *Bass*, *Baritone*). This range was used to present stimuli according to the selected range. *Post hoc*, we estimated that at least 60.59% of participants selected an appropriate range, based on matching the mean note they sang across all trials to the closest mean note of the different vocal ranges. This estimate is likely a lower bound, since vocal ranges somewhat overlap, and the mean singing note computed from trials is also dependent on the randomly selected melodies a participant heard.

#### Melodic stimuli set: The Berkowitz Corpus

Stimuli deployed by the SAA were drawn from Berkowitz et al. (2017; see also Baker, 2021), a corpus of melodies designed to improve sight-singing (singing from musical notation). The book consists of a collection of itemized melodic sequences. We took the first 629 sequences in the book and split them into N-grams^18^ of length 3 to 15. Based on evidence from the perceptual literature (Pembrook, 1987), we assumed 15 notes to cover the upper bound for short-term memory span of unknown melodies. We then removed any duplicate N-grams, resulting in a stimulus item bank of 159,127 unique N-grams.

#### Other tests and questionnaires

We now list the other tests and questionnaires utilized. To save space in the present manuscript, we keep the descriptions relatively brief, and encourage the reader to refer to the corresponding publications for more details. Some of the tests are adaptive. We refer the reader to Appendix Table 12 to see the reliability for the adaptive tests at the respective length we chose. Several have been comprehensively validated with respect to the item lengths we use (see Liu et al., 2023). We note that some reliabilities are fairly low, which might be due to characteristics of the sample used and the test with its concrete parameters as applied here. Overall, this suggests the use for more items per assessment in the future and the validation in a sample with a wide range of abilities.

##### Goldsmiths Musical Sophistication Index (Gold-MSI; Müllensiefen et al. (2014)

The *Goldsmiths Musical Sophistication Index* (*Gold-MSI*; Müllensiefen et al. 2014) is a self-report inventory for assessing dimensions of musicality in the general population. It was utilized here to produce scores of both musical training and singing abilities, based upon the Gold-MSI’s corresponding subscales of the same names, each comprising seven items 19. Higher amounts of musical training are hypothesized to be positively correlated with ability in singing, either because musical training facilitates the direct development of singing as a skill and/or the musicianship skills acquired through training (e.g., music theory, playing by ear) support the memory of musical content, such as melodies. Alternatively, those with more natural talent may be predisposed towards undertaking musical training, also producing a positive correlation (see Silas et al. 2022).

##### Melodic Discrimination Test (MDT)

Melodic discrimination ability was assessed using the adaptive *Melodic Discrimination Test* (*MDT*; Harrison et al. 2017). The test uses a 3-AFC response paradigm, with each item consisting of three versions of a melody played at different transpositions in pitch (for example: first: D major, second: Eb major, third: E major). Two of these versions are always identical and one is always different. The participant must identify the nonidentical melody, but ignore transpositions between versions. The ability to perceive and remember melodies well should serve as a predictor of our sung recall task, since to sing back melodies, one must first be able to remember them. Hence, better melodic discrimination ability should predict better sung recall performance. The version of the MDT used in this study comprised 11 items using an adaptive procedure (Harrison et al., 2017). IRT scores for the MDT task were generated online using the R package psychTestRCAT v1.0.2 (Harrison, 2018) according to the underlying IRT model described in (Harrison et al., 2017). The test utilized an adaptive procedure which adjusted to the ability of a participant based on a *psychTestRCAT* (Harrison, 2018) implementation.

##### Pitch Imagery Arrow Task (PIAT)

The *Pitch Imagery Arrow Task* (PIAT) has been established as a valid and reliable measure of musical imagery, the ability to mentally represent and transform pitch (Gelding et al., 2021). Participants must imagine going up and down a scale in relation to up and down arrows. They indicate whether their imagined tone was the same as a probe tone played at the end of a trial. A correct response requires identifying the correct place to end up in the scale based on the arrow indications. The ability to imagine musical content is a necessary component of sung recall. If one cannot imagine a melody they have heard after perceiving it, they will not be able to reproduce it. Hence, better pitch imagery ability should predict better singing performance. The task was adaptive with IRT scores for the PIAT task being generated online using the R package *psychTestRCAT* v1.0.2 (Harrison, 2018) according to the underlying explanatory IRT model (Gelding et al., 2021). There were 15 items. The test utilized an adaptive procedure that adjusted to the ability of a participant based on a *psychTestRCAT* (Harrison, 2018) implementation.

##### Pitch Discrimination Complex Tone Task (PDCT)

In a pitch discrimination complex tone task^20^, participants must discriminate the odd-one-out of a series of tones. There is a reference tone at a given audio frequency (here, 330 Hz), which may be at any point in the sequence, and other tones deviate from this frequency by varying amounts, with tones being closer to the reference tone being more difficult to detect. This perceptual pitch discrimination ability is a necessary part of the feedback mechanism to guide singing to be more or less in tune with target pitches, and hence, better pitch discrimination ability should predict singing ability. We re-implemented a complex pitch tone discrimination task with a 3-alternative forced choice (3-AFC) “odd-one-out” procedure like that described in Soranzo and Grassi (2014), but in the *psychTestR* environment^21^. The task was adaptive, and derived based on an IRT model we constructed using previously collected 3-AFC pitch discrimination data. There were 15 items. The test utilized an adaptive procedure which adjusted to the ability of a participant based on a *psychTestRCAT* (Harrison, 2018) implementation.

##### Mistuning Perception Test (MPT)

*The Mistuning Perception Test* (*MPT*; Larrouy-Maestri et al. 2019) is designed to assess whether a participant has the ability to detect whether a vocalist is singing “in-tune” against an audio track. Under a 3-AFC paradigm, participants must indicate which of three recordings contained an out-of-tune vocalist singing. The task of detecting whether a singer is in tune shows clear similarities to the task of monitoring one’s own singing and whether it is in tune. Hence, it is predicted that, as with the complex tone discrimination task, better mistuning perception ability should predict better singing ability. There were 15 items based on the adaptive version of the task and IRT model described in Larrouy-Maestri et al. (2019). The test utilized an adaptive procedure which adjusted to the ability of a participant based on a *psychTestRCAT* (Harrison, 2018) implementation.

##### Jack and Jill (JaJ)

Visuospatial working memory is broadly accepted to be a component of the wider construct of general working memory, which facilitates *all* cognitive tasks (Alloway & Alloway, 2013; Baddeley & Hitch, 1974). The *Jack and Jill* (*JAJ*; Tsigeman et al. 2022) task measures visuospatial working memory capacity based on a dual-task paradigm, similar to earlier versions of visuospatial dual-task paradigms (e.g., Alloway, Gathercole, Kirkwood, & Elliott, 2008; Shah & Miyake, 1996). Participants must hold multiple spatial locations on a hexagon in working memory while answering an unrelated question for each location point shown. Any cognitive task (of which singing is one) should necessarily involve some degree of working memory (Baddeley et al., 2009), thought to be “the” cognitive primitive (Alloway & Alloway, 2013; Silas et al., 2022). In the context of singing, it would underpin all cognitive aspects of the task, such as remembering a melody, reproducing it, and monitoring performance in real time. Difficulty of the task is primarily indicated as a function of item length, and hence, the ability to hold longer sequences in the task has an analogue to holding longer melodies in (musical) working memory. Therefore, higher working memory ability should predict better singing ability. IRT scores for the *JaJ* task were generated online using the R package *psychTestR* v 2.13.2 (Harrison, 2020) according to an underlying explanatory IRT model (Silas et al., 2022). There were eight items with the length of sequences increasing and hence becoming more difficult. The test utilized an adaptive procedure which adjusted to the ability of a participant based on a *psychTestRCAT* (Harrison, 2018) implementation.

### Procedure

All testing (i.e., both procedures A and B listed below) was conducted online, with participants completing the batteries at home on their own computers. Participants were told they would need headphones, a quiet room, and a microphone. Internal computer microphones were allowed. Each participant was asked to record: (1) a sample of their background noise by sitting in quietly for 5000 ms; (2) a note sung into their microphone for 5000 ms. These samples were used *post hoc* for signal-to-noise ratio (SNR) screening.

The main goal of the task was for a participant to sing back a note or melody which had been played to them. There were two main trial types: long note singing and melody singing. In long note trials, participants were presented a tone for 5000 ms and had to sing along with this tone immediately. All tones were presented in the participant’s selected range. A tone with similar parameters to the complex tone discrimination task in Soranzo and Grassi (2014) was used: a sine wave oscillator with four partials and envelope with a cosine attack curve and the following properties: attack: 0.01 s, decay: 0.01 s, sustain: 0.50 s (N.B. Soranzo and Grassi (2014) used 0.25 s), release: 0.01 s.

#### SAA: The rehearsal paradigm

SAA melody trials were designed to test not only singing accuracy but also newly learned melodic representations (i.e., generally corresponding to short-term memory/working memory^22^). Melodic stimuli were presented with a piano tone in a range that corresponded to the user’s specified range (e.g., Soprano, Alto), centered on the mean MIDI note of the stimuli. In melody trials, melodic stimuli were randomly sampled from the Berkowitz N-gram stimuli set we derived. Two possible melody trial types were deployed: rhythmic and arhythmic. In rhythmic trials, participants had to sing back a melody plus the rhythm it was presented with. In arhythmic trials, the rhythmic element was removed, and each note fixed to last 250 ms. The participant was encouraged to rehearse the melody aloud until they believed they had prepared it as best they could; the entire time, their output was recorded: hence, we called this the *rehearsal paradigm*. Participants clicked Stop to finish and could listen to the melody a maximum of three times per trial.

The originally intended function of the rehearsal paradigm was to observe the changes in patterns of sung recall across the temporal dimension of the trial (e.g., do N-gram chunks become more closely spaced throughout the rehearsal process?), and in particular, in a way that machine learning approaches could predict chunking patterns. However, this initial use case was discontinued, and the paradigm can here be thought of as providing a basis for a basic measure of accuracy: the number of notes recalled which were in the target stimulus. This accuracy measure captures two important properties of sung recall (Silas & Müllensiefen, 2023): 1) the number of notes recalled, which can reflect the general amount of effort expended by a participant (i.e., more notes, on the whole, = more effort) and 2) some indication as to the “level” of correctness (i.e., a higher proportion of notes being contained in the target stimulus = better performance). Note that the accuracy measure is only applied to the melody (and not the long note) trials described below. Also, all contents of the audio file reflecting a melody trial are analyzed with no pre-curation of which section is analyzed for analysis, hence (deliberately) leaving the possibility that some incidental vocal content is captured. Such “rehearsed” but incidental (incorrect) content should contribute to a lowering of the accuracy score, reflecting that the musical content is not yet retained in memory, hence reflecting (in)ability.

### Procedure A

One-hundred and seventy-five participants completed a short demographic questionnaire, six long note trials and then 15 arhythmic melody trials consisting of two trials of length 2 notes, and one trial each for lengths 3–15 and the same for rhythmic trials. Finally, they filled out the Gold-MSI Musical Training and Singing Abilities subscales. The procedure took 10 to 12 min.

### Procedure B

The remaining 72 participants completed the same procedure as the other participants, but in addition, the battery of additional tasks (PDCT, PIAT, MDT, MPT, JaJ). This alternative procedure took 30–40 min.

### Data analysis

Long note trials were not analyzed formally in Experiment 1, but were used for exploratory data analysis (not presented here); instead, Experiment 2 reports an analysis of long note data. The audio samples of a recorded background sound and the participant singing a long note were used to calculate a *post hoc* SNR for each participant. Participants whose SNR was < 0, reflecting a greater noise-to-signal (as opposed to signal-to-noise) ratio, were excluded from subsequent analyses (11 participants; ~ 220 trials), yielding 4,504 trials possible trials for analysis.

#### Audio Scoring

**Fig. 1:**
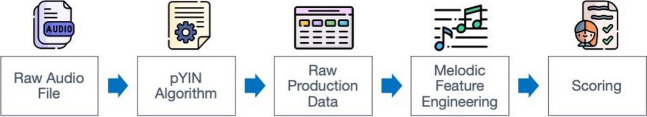
The pipeline from raw data to scored variables

Our data processing pipeline, which starts with the raw audio file and eventually yields meaningful scores, is summarized in Fig. 1. First, since the modern browser features we made use of only supported the *.webm* format (at least at the time), we converted all audio files to the *.wav* format^23^. Subsequently, audio files were processed in batch using the probabilistic YIN fundamental frequency estimation algorithm (*pYIN*; Mauch and Dixon (2014)), as hosted by the *Sonic Annotator* Vamp plugin (Cannam et al., 2010). This produced raw production data consisting of fundamental frequency estimates in Hz, the nearest MIDI pitch in the standardized Western tuning system, as well as each of the note’s corresponding temporal onset and duration estimates (see Cannam et al., 2010). These data were read into the *R* statistical programming environment where it was tidied and converted to useful symbolic representation formats (e.g., MIDI notes, musical intervals).

### Main analyses

The triaged sample of *N* = 236 was used for the construction of a mixed-effects model explaining participant performance on the singing tasks. In this experiment, we did not model rhythmic and arhythmic trials separately, but together in one model, described below. Later, for assessing individual differences in relation to other ability tests, only the smaller subset of *N* = 72, where the participants completed the larger battery of tasks, was used.

To serve as fixed-effect predictors, for each melodic stimulus, we computed the following melodic features, as described in Müllensiefen (2009): *i.entropy* (an estimate of the average level of “surprise” or randomness in musical interval^24^ information), *tonalness* (how strongly a melody correlates with a single key center), and *step.cont.loc.var* (the local variation in the contour [i.e., shape] of a melody)^25^. These were chosen due to previous research indicating that they could reflect melodic complexity and predict associated memory performance (Dreyfus et al., 2016; Harrison et al., 2016). Additionally, melody length was included as predictor, plus *d.entropy*, an estimate of the amount of “surprise” in rhythmic information, to indicate rhythmic complexity, and the log frequency of each melodic N-gram^26^ to indicate how more frequently occurring N-grams may be able to predict task performance (Pearce, 2018). See Appendix Table 13 for more information about the melodic features.

The dependent variable was called *proportion_of_correct_note_events*^27^. It was calculated as a proportion of “correct” notes (when rounded to the nearest integer MIDI pitch), to number of note events sung (a.k.a *precision* Silas & Müllensiefen, 2023). This is appropriate for the rehearsal paradigm, where the number of notes sung is expected to be considerably larger than the notes of the target melody, because we allowed rehearsal and multiple playback attempts, but recorded the entire sung recall in a single audio file.

A linear mixed-effects model with participant as random effect, *proportion_of_correct_note_events* as dependent variable, and the melodic feature predictors described above, as well as the categorical predictor *melody_type* (arhythmic vs. rhythmic) and its interaction with *d.entropy* (which would be related to *melody_type* across all melodies, but could vary differentially within each type) as fixed effects, was fitted to the data using the *R* package lme4 (Bates et al., 2015). From the resulting mixed-effects model, we extracted random intercept coefficients for each participant, which we took to represent a latent ability score on our new SAA task. We correlated this SAA score with scores of melodic discrimination ability (Harrison et al., 2017), mistuning perception ability (Larrouy-Maestri et al., 2019), pitch discrimination ability (Soranzo & Grassi, 2014) and visuospatial working memory ability (Tsigeman et al., 2022).

## Results

In the mixed-effects model, all seven melodic feature fixed-effect predictors were significant predictors of *proportion_of_correct_note_events*. See Table 1 for this model’s parameter estimates. As suggested, more local variation in a melody’s contour, tonalness, and whether a melody is rhythmic, are factors associated with a decrease the score. Conversely, a melody being more frequent in occurrence and having more surprise in musical interval or rhythmic information is associated with an increase in the score. The model mixed-effects *R*^2^ values (Nakagawa & Schielzeth, 2013) were: conditional *R*^2^c = .52 and marginal *R*^2^m = .20.

**Table 1:** Mixed-effects model with melody length (*N*), melody type (rhythmic vs. arhythmic), *step.cont.loc.var*, *tonalness*, *log.freq*, *d.entropy* and *i.entropy* as fixed effects and participant as random effect

Term	$\widehat{\beta }$	95% CI	$t$	$df$	$p$
Intercept	0.71	[0.65, 0.77]	22.96	3,272.78	< .001***
*N*	0.02	[0.02, 0.02]	10.04	3,406.01	< .001***
Step cont loc var	– 0.28	[– 0.34, – 0.22]	– 8.60	3,406.67	< .001***
Tonalness	– 0.11	[– 0.16, – 0.06]	– 4.39	3,405.52	< .001***
Log freq	0.01	[0.00, 0.01]	2.27	3,418.09	.023*
I entropy	0.25	[0.11, 0.38]	3.49	3,416.53	< .001***
Melody typeTRUE	– 0.43	[– 0.47, – 0.38]	– 18.62	3,441.11	< .001***
Melody typeTRUE $\times$ D entropy	0.30	[0.01, 0.58]	2.03	3,417.43	.043*

*p* < .05*, *p* < .001***

## Bivariate correlations with other individual differences measures

Utilizing the data subset (*N* = 72), which measured user performance on several other tasks, we assessed how SAA ability scores might be related to other individual differences measures. The SAA score we derived demonstrated statistically significant correlations with all measures except for the measures of visuospatial working memory and pitch discrimination. It had small to moderate positive correlations with melodic discrimination, pitch imagery abilities, a large positive correlation with mistuning perception ability and moderate positive correlations with self-reported singing ability and musical training. See Table 2 for the Pearson’s correlation values.

**Table 2:** Pearson’s correlations of dependent variables in Experiment 1

	1	2	3	4	5	6	7	$\mathrm{M}$	$\mathrm{SD}$
1. JAJ.ability								0.84	0.70
2. MDT.ability	.24*							1.14	0.96
3. MPT.ability	.21	.39***						0.93	0.77
4. PIAT.ability	.35**	.43***	.41***					1.73	1.69
5. PDCT.ability	.25*	.23*	.45***	.26*				0.40	0.36
6. GMS.SA	.41**	.50***	.55***	.46***	.39**			5.31	1.39
7. GMS.MT	.06	– .06	.39**	.16	.06	.40**		5.10	1.05
8. SAA_Ability	.15	.28*	.63***	.45***	.19	.41**	.43***	0.12	0.08

*p* < .05*, *p* < .01**, *p* < .001***

## Discussion

In Experiment 1, we developed a prototype singing test for online data collection. We then undertook audio frequency and note onset estimation and scoring procedures *post hoc* and modeled the resulting data at the level of the individual trial. We were able to create a statistically significant explanatory IRT model which explained a moderate (20–52%) proportion of variance in the data. Especially given the data’s “dirty” nature, this can be considered a successful result. This result supports our hypothesis that features which indicate melodic complexity (including melody length) are relevant predictors, offering explanatory power in accordance with the previous literature (Dreyfus et al., 2016; Fleurian et al., 2017; Müllensiefen & Halpern, 2014). Moreover, the relatively large difference between the marginal and conditional *R*^2^ values suggests that there is a sizeable proportion of individual differences in the sample of participants tested which explains SAA performance. This is in line with our predictions, that individual differences should explain performance on a task in which one can develop high levels of domain-specific expertise.

To investigate the nomothetic span (i.e., the network of relationships of a test score with other variables; Whitely 1983) of the SAA with potentially related abilities, we assessed how the derived SAA score was related to other individual difference measures by extracting random effects coefficients for each participant based on the derived mixed-effects model. The random effects coefficients were taken to represent a latent melodic singing ability (SAA) score. In line with theories of singing accuracy (Pfordresher et al., 2015), sung recall was related to melodic discrimination, pitch imagery abilities, and mistuning perception, which seem to be constituent, lower-level abilities that contribute to the higher-level skill of melodic sung recall. This can offer concurrent validity to our derived SAA score. In this way, the SAA score was also moderately correlated with self-reported singing ability (*r* = .46) which bolsters its validity and plausibility further, and is comparable to similar correlations reported by singing research conducted online (e.g., Tan et al., 2021). However, the SAA score showed non-significant correlations with visuospatial working memory and pitch discrimination abilities. This suggests that singing ability may not be very closely related to low-level perceptual processes or non-musical working memory capacity, at least in the way the SAA task was presented and scored here, using the rehearsal paradigm as experimental task and *proportion_of_correct_note_events* as dependent variable. The null correlation between singing and pitch discrimination abilities has actually been observed in previous research (e.g., Pfordresher & Brown, 2007), which can also provide validity to the SAA, in that it replicates previous research results. Lastly, the SAA score was related to musical training, which suggests that singing abilities may be improved by musical training. However, the reverse causal explanation could also be true: those with already good singing abilities may be more likely to undertake more musical training (see Silas et al., 2022) for a discussion of such issues of causality in musical training).

Importantly, considering the issue of “dirty musical data”, our new task and analysis pipeline seems to produce consistent results. However, several improvements could be made. In this respect, Experiment 2 describes the development of several new features: in particular, the computation of a signal-to-noise ratio on-the-fly, as well as the replacement of the rehearsal paradigm with a new procedure that enables a more efficient deployment of the SAA. In particular, whilst the rehearsal paradigm, scored with a measure of accuracy, appears to hold good validity, as indicated by its directional relationships with other relevant ability tests, in a more comprehensive study profiling accuracy vs. similarity measures on singing data, we found accuracy measures to hold notable limitations (Silas & Müllensiefen, 2023). For instance, accuracy measures do not take the order of recalled notes into account, which is important musically. For this, and other reasons profiled in Silas and Müllensiefen (2023), we proceed in Experiment 2 with similarity metrics for scoring sung recall data.

## Experiment 2: Validation of the SAA “one-shot” paradigm

The overarching objective of Experiment 2 was to update the SAA task to be more sophisticated and prepared for adaptive testing (Harrison, 2018) in the future. First, the audio data processing undertaken *post hoc* in Experiment 1 was now intended to work in real time, as the test progresses. Second, to make the test more efficient, we decided to discontinue the rehearsal paradigm, which yields long patterns of rehearsed sung recall, and replace it with the “one-shot” paradigm, whereby the participant hears a melody once, and must sing it back immediately, without rehearsal. To capture how learning develops over time, instead of capturing rehearsal, we may instead either allow a single attempt or multiple attempts, each with a new, distinct audio recording of the “one shot”.

Third, in view of this new paradigm, we employed a new main dependent variable, *opti3*, an established measure of melodic similarity (Müllensiefen & Frieler, 2004a, 2007; Pearce & Müllensiefen, 2017; Silas & Müllensiefen, 2023). *opti3* is a hybrid measure derived from the weighted sum of three individual measures which represent different aspects of melodic similarity. The similarity in interval content is captured by the *ngrukkon* measure that measures the difference of the occurrence frequencies of short pitch sequences (*N-grams*) (e.g., length 3–8) contained within two melodies (Uitdenbogerd, 2002). Harmonic similarity is measured by the *harmcore* measure. This measure is based on the chords implied by a melodic sequence, taking pitches and durations into account. Implied harmonies are computed using the Krumhansl–Schmuckler algorithm (Krumhansl, 1990) and the harmonic sequences of the two melodies are compared by computing the number of operations necessary to transform one sequence into the other sequence (i.e., the so-called edit distance; Mongeau and Sankoff 1990). Finally, rhythmic similarity is computed by first categorizing the durations of the notes of both melodies (known as “fuzzification”) and then applying the edit distance to measure the distance between the two sequences of durations. The resulting measure of rhythmic similarity is called *rhythfuzz* (Müllensiefen & Frieler, 2004a)^28^. See Appendix Table 14 for more information and for an even more comprehensive explanation of how these measures work on sung recall data, with intuitive examples, we refer the reader to our other research (Silas & Müllensiefen, 2023). Based on the perception data collected by Müllensiefen and Frieler (2004a), the three individual measures are weighted and combined to form a single aggregate measure of melodic similarity, *opti3*:

1 $$opti3=3.027*ngrukkon+2.502*rhythfuzz + 1.439*harmcore$$

Hence, *opti3* is sensitive to similarities and differences in three important aspects of melodic perception (pitch intervals, harmonic content, rhythm). We note that all three individual measures (*ngrukkon*, *harmcore*, *rhythfuzz*) can take values between 0 (= no similarity) and 1 (= identity) and are length-normalized by considering the number of elements of the longer melody. It is particularly appropriate for the one-shot paradigm because it allows the computation of similarity between a target melody and a sung recall which may differ slightly, but not greatly, in length. Moreover, unlike the dependent variable, *proportion_of_correct note_events* from Experiment 1, *opti3* observes the order of note events which is an important feature of melodies. See Appendix Table 14 for descriptions about these variables and Silas and Müllensiefen (2023) for a comprehensive assessment of melodic similarity measures applied to sung recall data.

Fourth, we aimed to implement additional lower-level note and melody singing-based measures (e.g., interval precision, note accuracy), as presented in the singing accuracy literature (Pfordresher et al., 2010), rather than those which deal solely with melodic similarity. Consequently, fifth, Experiment 2 also formally models long note singing ability, taken to represent a lower-level singing ability when compared to melodic singing ability. Lastly, we aimed to add other features to improve the quality of the data collected by the online test interface, as well as adding feedback features, so that eventually such a test could be readily expanded for use in educational settings (see Table 11 for an overview of the features).

With regards to item response theory modeling, we hypothesized that the modeling of arhythmic and rhythmic melodic singing data might require different statistical models. Each distinct model and respective trial blocks should serve as distinct outputs for use by other researchers, depending on their research questions and requirements.

## Method

### Singing Ability Assessment (SAA) enhancements

As a first step in upgrading our task, we made all *post hoc* steps taken in Experiment 1 (e.g., determining the SNR, processing audio files, scoring the data, etc.) to be now available at test time. In addition, several new features were added to the processing chain of collecting and analysing sung recall data. We describe two important updates in detail below, although inspecting the arguments to the main SAA function in the *R* package of the same name^29^ will provide a comprehensive list.

#### Real-time signal to noise ratio (SNR) computation

In Experiment 1, the signal-to-noise ratio (*SNR*) was determined *post hoc* and participants disqualified then. This is inefficient, since some participants complete the test despite having bad SNRs. Consequently, we designed an SNR test which works at test time and can optionally disqualify participants who did not reach a specified threshold^30^. The SNR formula consists of computing the ratio of the signal amplitude over the background noise amplitude. These amplitudes can be estimated with the root mean square, and the SNR is calculated in *dB* according to

2 $$SNR=20\times \mathrm{log}10\left(RM{S}_{signal}/RM{S}_{noise}\right)$$

Whereas in Experiment 1, we used the SNR value of 0, we found a more principled selection based on Kim et al. (2018). The graphs in their paper suggested that the *pYIN* algorithm’s accuracy starts deteriorating substantially when an SNR ratio < 14 is present. Consequently, by default, all participants are required to have a minimum SNR of 14 to proceed with the rest of the SAA test.^31^

#### Real-time vocal range determined from singing

Instead of participants selecting a vocal range which best suits their voice based on audio examples, the new version of the test asks the participant to sing a low note and a high note, and based on this, computes a vocal range, or a likely vocal range^32^. After the individual vocal range has been captured, each stimulus will be transposed into the range of the participant such that its mean note is matched to the mean note of the user’s range.

### Participants

A total of 910 participants aged 16–72 (*M* = 31.07, *SD* = 11.54; 66.22% female were recruited through the *SliceThePie* marketing panel, across four testing conditions (*N* = 219; *N* = 249; *N* = 207; *N* = 227); 67% were from the US, 25% UK, 5% Canada, and the remaining other countries. Eight participants’ demographic data was missing (reason unknown).

### Materials

Other than the updated SAA test, the only other material employed was the *Gold-MSI* inventory as described in Experiment 1. This again yielded self-reported measures of Musical Training and Singing Abilities based on the factor model described in Müllensiefen et al. (2014). The task was again deployed on an *AWS EC2* server instance, where the scoring was now done in real-time. All scores were downloaded *post hoc*for statistical analyses.

### Procedure

The procedure of the SAA battery was essentially the same as Experiment 1, but with scoring being done on-the-fly (not known to the participant), as well as the SNR test disqualifying people at test time, and the vocal range being computed in real time via singing low and high notes. The long note singing task was also identical, except for the new scoring measures computed at the backend of the test.

#### The one-shot paradigm

In Experiment 1, participants were encouraged to rehearse learning a melody aloud, and could hear a target melody up to three times during their rehearsal process. Consequently, each audio file might represent up to three distinct attempts (i.e., after each playback), as well as rehearsal within/between each discrete attempt.

Conversely, the melody singing paradigm in Experiment 2 required participants to sing back a melody in ‘one shot’ after hearing it. The meaning of one-shot here means “without rehearsal” and that, after hearing a melody, the participant must try sing it back as best they can immediately (once). This produces a clear one-to-one correspondence between a heard melody, a sung recall, and an audio file. However, as in Sloboda and Parker (1985), there can still be multiple attempts per item (by default, up to 4, for statistical reasons). The difference is that the one-shot paradigm produces one audio file per attempt, unlike in the rehearsal paradigm, where multiple distinct attempts might all be contained in one audio file. In both cases, attempts are nested in items; in the rehearsal paradigm, all attempts and rehearsal are nested in a single audio file; in the one-shot paradigm; each single attempt is in a single audio file.

#### Procedure variants

Testing was deployed across four different conditions, which were released online via *SliceThePie* in a staggered fashion, but then ran simultaneously: (1) one-attempt arhythmic melodies; (2) one-attempt rhythmic melodies; (3) multi-attempt-arhythmic melodies; (4) multi-attempt rhythmic melodies. In the multi-attempt variants, participants could optionally have up to three attempts per melody, if they wanted.

### Data analysis

A summary of the variables computed from the raw data and used across the experiments is presented in Table 3.

**Table 3:** Variables used across the experiments, arranged by category: Long Note, Melody, Established measures of singing accuracy, and hardware

Measure	Description
Long Note	
long_note_accuracy	The average deviation from the target note in cents.
long_note_var	The variance of the *pYIN* smoothed pitch track (in Hz).
long_note_dtw_distance	The distance between an idealized pitch track and the sung pitch track, as computed by the dynamic time warp algorithm.
long_note_autocorrelation_mean	The mean autocorrelation value of the pYIN smoothed pitch track (in Hz).
long_note_run_test	The Wald–Wolfowitz runs test statistic applied to the pYIN smoothed pitch track (in Hz).
long_note_no_cpts	The number of 'changepoints' as computed by the cpt.mean function from the R package changepoint
long_note_beginning_of_second_cpt	The beginning of the second changepoint in seconds (which could indicate long note scoop).
pca_long_note_accuracy	A PCA-weighted sum comprised predominantly of long_note_accuracy and long_note_dtw_distance.
pca_long_note_volatility	A PCA-weighted sum comprised predominantly of long_note_autocorrelation_mean, long_note_run_test and long_note_no_cpts.
pca_long_note_scoop	A PCA-weighted sum comprised predominantly of long_note_no_cpts and long_note_beginning_of_second_cpt.
Melody	
SAA_Ability	A score reflecting ability on both arrhythmic and rhythmic items simultaneously. It is equivalent to the random participant intercept from Model 1.
SAA_Ability_Arrhythmic	A score reflecting ability on only arrhythmic items. It is equivalent to the random participant intercept from Model 2.2
SAA_Ability_Rhythmic	A score reflecting ability on only rhythmic items. It is equivalent to the random participant intercept from Model 3.2
* opti3*	A hybrid measure of melodic similarity comprising a weighted sum of the similarity of interval, rhythm, and harmonic information (Müllensiefen & Frieler, 2004a, b). Specifically: opti3 = 3.027 * ngrukkon + 2.502 * rhythfuzz + 1.439 * harmcore.
proportion_of_correct_note_events	The proportion of correct note events ("correct" meaning "contained in stimulus"), as sung by the user.
Established measures of singing accuracy	
melody_note_precision	The consistency with which a singer produces specific pitch classes across repeated occurrences, independent of the proximity of each occurrence to the target pitch. (Pfordresher et al. 2010)
melody_note_accuracy	Average proximity of each produced F0 to each target F0 (Pfordresher et al. 2010).
interval_precision	A similar measure to note precision, but for intervals. (Pfordresher et al. 2010)
interval_accuracy	A similar measure to note accuracy, but for intervals. (Pfordresher et al. 2010)
pca_melodic_singing_accuracy	A PCA-weighted sum comprising of melody_note_precision, interval precision and interval_accuracy.
Hardware	
hardware_concurrency	The number of logical processors available to run threads on the user's computer.
device_memory	The approximate amount of device memory in gigabytes and the self-reported indicator of whether a user was using an internal or external microphone.

#### Long note singing

To analyze the long note data, first we averaged the scores across the five trials, for each participant, on each of the seven long note singing measures as described in Table 3. Then we employed parallel analysis (Horn, 1965) and a series of principal components analyses (PCA) as a means of dimension reduction. Long note scores were extracted for each participant from the final PCA model. This score was taken to represent a basic low-level note singing ability, distinct from melodic singing.

#### Melody singing

The melody singing analysis was much the same as Experiment 1, employing the explanatory item response theory modeling approaches described earlier, but with *opti3* as dependent variable. In addition to a model which models all data (rhythmic and arhythmic) simultaneously, yielding an overall *SAA_Ability_Score*, we create separate models based on only arhythmic (*SAA_Arhythmic*) or rhythmic (*SAA_Rhythmic*) melodic data. Later in our analyses, we use the broader *SAA_Ability_Score* for relating rhythmic and arhythmic melody data to other variables simultaneously, though we recommend that the two separate arrhythmic (*SAA_Arhythmic*) and rhythmic (*SAA_Rhythmic*) scores are used by future researchers, to reflect the slightly distinct abilities which the models represent. The empirical dataset comprised 7145 trials of data, with 5580 unique melodic items selected from the tokenized (N-gram) Berkowitz corpus.

Initially we had planned to also analyze the multiple attempt versions of our data collection separately and include attempt as a fixed effect. However, very few participants actually elected to take a second or third attempt. While there are 6633 trials of participants having a first attempt, there are only 417 for a second attempt and 95 for a third attempt. This seems to suggest that multiple attempt trials do not seem to work well in the context of a relatively uncontrolled Internet experiment, at least when there is no incentive for participants to increase their singing accuracy. Consequently, we did not model attempt and instead, filtered the dataset to only contain the first trial (i.e., even where participants could have had more than one attempt).

#### Principal components analysis of established measures of melody singing accuracy

Instead of assessing the relationship of the derived *SAA_Ability_Score* through correlations with other musical ability tests as in Experiment 1, we assessed it alongside previously validated measures of singing accuracy described in Pfordresher et al. (2010) (note accuracy, note precision, interval accuracy, interval precision), scored on the same data. However, first we submitted these variables to a unidimensional PCA and extracted component scores for each sung melody from the resulting model.

#### Higher-level modeling

These dimension reduction processes yielded aggregate melodic singing scores, along with the aggregate long note component scores, which we then correlated to assess their relationship with one another. Additionally, we assessed the relationship of the *SAA_Ability_Score* derived from the explanatory item response model (i.e., random intercepts from the mixed-effects model) which includes both rhythmic and arhythmic melodies with measures of hardware setup which were collected through the Internet browser, as a means of determining potential error sources. The hardware measures were *hardware_concurrency*, defined as the number of logical processors available to run threads on the user’s computer, and *device_memory*, the approximate amount of device memory in gigabytes, and the self-reported indicator of whether a user was using an internal or external microphone.

Finally, to formally model how lower-level singing abilities as well as demographic predictors (age, gender, level of musical training) might predict the higher-level melodic recall *SAA_Ability_Score*, we constructed a multiple regression model with the *SAA_Ability_Score* as dependent variable and the lower-level variables (e.g., *note_precision*, *long_note_accuracy*) described above as predictors.

## Results

### Long note singing

When submitting the long note variables to a parallel analysis, three components were suggested. Consequently, a three-dimensional PCA was fit to the long note data. In the solution, all indicators had a communality (h^2^) value above .75, except for *long_note_var*. This was removed and a second three-dimensional PCA was fitted. In this solution (see Table 4), all h^2^ values were above .75. Each indicator had a factor loading of at least .5, with each component explaining a cumulative proportion of 30, 59, and 85%. The first component seemed to represent volatility in pitch frequency (i.e., the tendency for the pitch curve to be erratic, rather than stable), the second, general long note accuracy, and the third, “scooping” or change points to the sung note. Note that *long_note_no_cpts* cross loads onto the accuracy and scooping components. Component scores were extracted for each participant on each of the three latent variables.

**Table 4:** Final principal components analysis solution for long note data

Variable	RC1	RC2	RC3	h2	u2
long_note_accuracy	– 0.04	0.93	– 0.06	0.87	0.13
long_note_dtw_distance	0.20	0.88	0.11	0.83	0.17
long_note_autocorrelation_mean	0.84	0.19	– 0.18	0.77	0.23
long_note_run_test	– 0.90	0.02	0.00	0.81	0.19
long_note_no_cpts	0.52	0.05	– 0.78	0.88	0.12
long_note_beginning_of_second_cpt	0.05	0.07	0.95	0.92	0.08

### Melody singing

To assess the relative differences between arhythmic and rhythmic trial types, and hence to decide whether separate models for arhythmic vs. rhythmic trial types are warranted, our first mixed-effects model (Model 1) modeled all (i.e., arhythmic and rhythmic) data simultaneously. *opti3* was dependent variable, *N step.cont.loc.var*, *tonalness*, *log_freq*, *d.entropy*, *melody_type* (arhythmic vs. rhythmic) and the interaction of *melody_type* with *d.entropy* were fixed effects and participant was used as a random intercept effect. In the model (see Table 5), all fixed-effect predictors were significant, except the effect of *d.entropy* within the condition of *arhythmic*, which is to be expected, considering that rhythmic variability is not present in arrhythmic melodies. The *R*^2^m value was .16 and the *R*^2^c value was .42, suggesting that the model explained a moderately large amount of the variance in the data, with the fixed effects along explaining a small amount of variance in the data. The coefficient of *melody_type* was $B$ = – .15 (*p* < .001), suggesting that rhythmic trials are associated with a higher difficulty. This suggests that arhythmic and rhythmic trials should be modeled separately, by being somewhat categorically different in difficulty.

**Table 5:** Model 1: Mixed-effects model regressing SNR onto melodic feature variables as fixed effects and participant as random effect, across all melodic stimulus items

Term	$\widehat{\beta }$	95% CI	$t$	$df$	$p$
Intercept	0.64	[0.58, 0.70]	21.39	6,940.88	< .001***
*N*	– 0.01	[– 0.01, – 0.01]	– 6.42	6,797.10	< .001***
Step cont loc var	– 0.38	[– 0.45, – 0.32]	– 11.91	6,701.28	< .001***
Tonalness	0.10	[0.06, 0.15]	4.39	6,611.36	< .001***
Log freq	0.01	[0.01, 0.01]	5.74	6,638.89	< .001***
Melody typerhythmic	– 0.15	[– 0.18, – 0.11]	– 8.05	6,887.32	< .001***
Melody typearrhythmic $\times$ D entropy	– 0.08	[– 0.16, 0.01]	– 1.79	6,636.10	.073
Melody typerhythmic $\times$ D entropy	– 0.28	[– 0.41, – 0.16]	– 4.46	6,646.15	< .001***

*p* < .001***

Next, a similar model (Model 2.1) was specified, but only for arhythmic trials, and hence, the *melody_type* (arhythmic vs. rhythmic) factor was not included. *d.entropy* and *i.entropy* were not significant predictors and were removed. In the resulting arhythmic model (Model 2.2), *N*, *step.cont.loc.var*, *tonalness* and *log_freq* were significant. The *R*^2^c was .38 and the *R*^2^m was .11. See Table 6.

**Table 6:** Model 2.2: Mixed-effects model regressing opti3 onto melodic feature variables as fixed effects and participant as random effect, with only arhythmic trials

Term	$\widehat{\beta }$	95% CI	$t$	$df$	$p$
Intercept	0.74	[0.66, 0.82]	17.61	3,104.92	< .001***
*N*	– 0.01	[– 0.01, 0.00]	– 2.68	3,124.27	< .01**
Step cont loc var	– 0.32	[– 0.41, – 0.24]	– 7.66	3,062.54	< .001***
Tonalness	0.13	[0.07, 0.19]	4.02	2,983.24	< .001***
Log freq	0.02	[0.01, 0.02]	6.16	2,960.72	< .001***

*p* < .01**, *p* < .001***

The same process was undertaken to model only rhythmic melody trials. In the resulting model (Model 3.2), *N*, *step.cont.loc.var*, *log_freq*, *d.entropy* and *i.entropy* were significant predictors. The *R*^2^c was .42 and the *R*^2^m was .13. See Table 7.

**Table 7:** Model 3.2: Mixed-effects model regressing SNR onto melodic feature variables as fixed effects and participant as random effect, with only rhythmic trials

Term	$\widehat{\beta }$	95% CI	$t$	$df$	$p$
Intercept	0.37	[0.32, 0.42]	14.27	3,283.70	< .001***
*N*	– 0.01	[– 0.01, 0.00]	– 2.81	3,143.74	< .01**
Step cont loc var	– 0.50	[– 0.64, – 0.35]	– 6.80	3,096.88	< .001***
Log freq	– 0.02	[– 0.02, – 0.01]	– 4.60	3,061.91	< .001***
D entropy	– 0.26	[– 0.38, – 0.14]	– 4.42	3,062.86	< .001***
I entropy	– 0.19	[– 0.33, – 0.04]	– 2.56	3,056.75	.010*

*p* < .05*, *p* < .01**, *p* < .001***

Random effects coefficients for participant were extracted from the three different models, which had (1) *melody_type* (arhythmic vs. rhythmic) as a fixed-effects predictor, as well as the resulting (2) arhythmic vs. (3) rhythmic models. These were taken to represent three distinct ability scores (*SAA_Ability*, *SAA_Ability_Arrhythmic* and *SAA_Ability_Rhythmic*). Note that the *SAA_Ability* score is modeled on the same data as the *SAA_Ability_Arrhythmic* and *SAA_Ability_Rhythmic* ability scores, but modeling the data they were built with simultaneously.

The models constructed above can be used to compute item difficulty scores for any melody in the Berkowitz corpus. This allows the creation of an adaptive (and hence efficient) test via the *R* package *psychTestRCAT*, which re-estimates participant ability after each trial, based on the current item’s difficulty value. We computed difficulty values for all items in the Berkowitz corpus of melodies, which is released as a separate item bank in the Berkowitz package^33^. These difficulty values are essentially a model prediction (where *opti3* is the dependent variable), given the fixed-effects values for each melody in the corpus (i.e., it is an output of the sum of the fixed-effects values for each melody, weighted by the fixed-effects coefficients described in this paper).

### Principal components analysis of established measures of melody singing accuracy

The variables *note accuracy*, *note precision*, *interval accuracy* and *interval precision* were submitted to a unidimensional PCA. In the solution, all indicators were at a communality (h^2^) value above .30, except for *melody_note_accuracy*. This was removed and, in the final solution (see Table 8), note precision, interval precision and melody interval accuracy had factor loadings above .50 and h^2^ values above .4. The single factor achieved to explain 51% of variance in the data. Components scores were extracted from this model, and we called the new aggregate variable *pca_melodic_singing_accuracy*.

**Table 8:** Final principal components analysis solution for melody singing accuracy data

Variable	PC1	h2	u2
note_precision	0.82	0.67	0.33
interval_precision	0.64	0.41	0.59
melody_interval_accuracy	0.66	0.44	0.56

### Higher-level modeling

The correlations among the continuous variables are shown in Table 9. As shown, there are a range of correlation magnitudes from null to moderate, which tend to vary by group: the self-report questionnaires have a moderate correlation with one another, but only small or no correlations with other variables; the three SAA scores we derived from the models constructed from rhythmic, arhythmic and all models have large correlations with one another. In summary, the table shows that most variables are related to some degree, but there is no multicollinearity, suggesting a good balance of convergent vs. divergent validity.

**Table 9:** Pearson’s correlations of dependent variables in Experiment 2 (Holm’s corrected)

	1	2	3	4	5	6	7	8	9	10	$\mathrm{M}$	$\mathrm{SD}$
1. Self-reported Musical Training											3.27	1.40
2. Self-reported Singing Abilities	.50***										4.31	1.01
3. SAA_Ability	.24***	.25***									0.00	0.11
4. SAA_Ability_Arrhythmic	.24***	.21***	.95***								0.00	0.11
5. SAA_Ability_Rhythmic	.27***	.29***	.94***	.72***							0.00	0.10
6. hardwareConcurrency	.02	– .06	– .05	– .04	– .08						6.07	3.46
7. deviceMemory	.00	– .10	.09	.12	.05	.39***					6.45	2.16
8. pca_long_note_volatility	– .07	– .08	– .03	.04	– .05	.11	.13*				0.07	0.91
9. pca_long_note_accuracy	– .15***	– .09	– .23***	– .24***	– .23***	– .05	– .06	– .03			0.01	1.02
10. pca_long_note_scoop	– .08	– .05	– .08	– .04	– .12	.01	.02	.11*	.01		– 0.04	0.94
11. pca_melodic_singing_accuracy	– .20***	– .25***	– .56***	– .50***	– .60***	.10	.04	.10	.19***	.06	– 0.01	0.99

*p* < .05*, *p* < .001***

In the higher-level multiple regression model with the main *SAA_Ability_Score* (i.e., derived from rhythmic and arhythmic melody simultaneously) as dependent variable, the demographic variables *Musical Training*, *Age* and *Gender*, the long note singing variables *pca_long_note_volatility*, *pca_long_note_accuracy*, *pca_long_note_scoop* were used as predictors as well as the variables that were excluded from the PCA models, namely *pca_long_note_randomness*, *pca_long_note_scoop*, *long_note_var* and *melody_note_accuracy*. The predictors *pca_long_note_volatility*, *pca_long_note_scoop* and *long_note_var* made no significant contribution to the model and were therefore removed as predictors. The final model had an *R*^2^ value of .38 (adjusted *R*^2^ = .37), *p* < .001, and is shown in Table 10.

**Table 10:** Regression model with the SAA score as dependent variable and lower-level singing variables as predictors. Variables were standardized before model fitting to make small unstandardized beta estimates more interpretable

Predictor	$b$	95% CI	$t$	$df$	$p$
Intercept	0.57	[0.50, 0.64]	16.27	815	< .001***
Musical Training	0.07	[0.03, 0.10]	3.57	815	< .001***
Age	– 0.05	[– 0.09, – 0.01]	– 2.25	815	.024*
GenderMale	– 0.03	[– 0.05, – 0.01]	– 3.27	815	< .001***
Pca long note accuracy	– 0.15	[– 0.23, – 0.07]	– 3.57	815	< .001***
Pca melodic singing accuracy	– 0.51	[– 0.57, – 0.45]	– 16.85	815	< .001***
Melody note accuracy	0.23	[0.12, 0.34]	4.15	815	< .001***

*p* < .05*, *p* < .01**, *p* < .001***

The size and direction of the coefficients are in line with expectations, considering that some of the singing accuracy scores (e.g., *pca_melodic_singing_accuracy*) reflect error (i.e., a smaller error score can predict a better *SAA_Ability_Score*).

## Discussion

The main objective of Experiment 2 was to implement the beginning steps of creating an adaptive singing test. Firstly, this required giving the static test developed in Experiment 1 new features, which for example, compute results (from fundamental frequency and note onset information through to psychometric scores) on-the-fly.

We also formally modelled the long note data, which suggested that there are different aspects of single-note singing ability which can be reflected in the data. These features seem to represent the level of volatility, general accuracy and the scoop or number of changes in the fundamental frequency pitch curve. For melody singing trials, we updated the paradigm from the so-called rehearsal paradigm to the new one-shot paradigm. This latter paradigm produces cleaner data and is generally easier to work with, since it produces one iteration of a sung recall per audio recording. For this paradigm, we chose *opti3* (Müllensiefen & Frieler, 2004a), a measure of melodic similarity, as the main dependent variable. We view *opti3* scores as measures of an overall melodic recall ability which reflects both melodic memory accuracy and singing accuracy. Use of the one-shot paradigm allowed us to separate multiple attempts at the same item into distinct audio files. However, it was observed that only a small proportion of participants were willing to optionally expend the extra effort to take multiple attempts. This effect of effort is a problem for all performance research (Silm et al., 2020), but is particularly difficult or impossible to control in the context of an online experiment. This suggests that researchers should be careful overextrapolating from results collected online, but also demonstrates the need to minimize test lengths where possible (e.g., through adaptive testing).

As a means of determining divergent and construct validity, we compared model outputs built with *opti3* with other related measures such as self-reported musical training, and additionally, implemented several melody singing accuracy measures described in the previous literature (Pfordresher et al., 2010). Small statistically significant positive correlations with self-reported singing accuracy and musical training are in line with expectations. As expected, certain objective indicators of singing accuracy seem to predict a portion of the variance in *opti3* scores. The established melodic singing accuracy measure variables in our regression model had substantial (${\beta }_{pca\_melodic\_singing\_accuracy}=- 0.51$, *p* < .001; ${\beta }_{melody\_note\_accuracy}=0.23$, *p* < .001) standardized magnitudes^34^, suggesting that low-level singing accuracy is predictive of the overall *opti3* construct, which we suggest represents variance in melodic memory also. The standardized coefficient on *pca_long_note_accuracy* was even smaller (${\beta }_{pca\_long\_note\_accuracy}=- 0.15$, *p* < .001), suggesting that even the ability to sing distinct stable tones is a factor in overall sung recall. However, these measures are not highly related or colinear, suggesting that some proportion of variance may be to do with melodic memory, beyond singing accuracy.

Broadly speaking, the results in Experiment 2 suggest that long note singing and melodic singing are somewhat differentiated, as indicated by the PCA models, suggesting they are relatively distinct tasks. This is most likely because long note singing does not involve sophisticated mental templates of melodic structure and is more about fine-grained pitch production monitoring. In other words, long note singing depends more on simple low-level perceptual processes and less on high-level learned representations.

Lastly, certain demographic features were related to the overall *SAA_Ability_Score*: ${\beta }_{MusicalTraining}=0.07$ (*p* < .001); ${\beta }_{Age}=- .05$ (*p* = .02); ${\beta }_{GenderMale}=-.03$ (*p* < .001), but with relatively small effects, such that: more musical training predicts better SAA ability, a lower age predicts a better *SAA_Ability_Score*, and women performed better than men. The latter two effects are particularly small and could be to do with idiosyncrasies in the sampling panel we used, so we do not extrapolate too much from them.

A next step for obtaining reliability and validity of our analysis procedure is to compare the automated *pYIN* transcription of sung recall and subsequent *opti3* scoring results to those produced when using transcriptions by a professional human rater, on the same data. We have conducted such an experiment, but it is beyond the scope of the present paper and will instead be presented in a forthcoming publication. However, preliminary results show that the mean edit distance accuracy between the *pYIN* output with default parameters settings and professional human transcription was 65%, but improved to an edit distance accuracy of 73% after optimizing the *pYIN* parameters (see Müllensiefen and Frieler (2007) for a description of edit distance applied to musical data). This suggests the automated transcription procedure is not perfect, but also corresponds largely to human professional transcription.

Additionally, we showed that participants’ hardware features are related to sung recall performance. Note that this does not prove a causality: there are at least two opposing causal explanations. For example: i) a poorer hardware setup decreases sung recall performance through creating latency/test presentation issues which interferes with the participant’s performance vs. ii) those with higher socioeconomic status can afford better hardware setups and coincidentally have more training/higher cognitive abilities. These possibilities can both be simultaneously true and contribute to the relationship and must be explored more in future research. However, as far as we are aware, we are the first to document such a relationship in Internet singing research.

To create a prototype computerized adaptive test based on psychometric scoring, we constructed mixed-effects models separately for performance on rhythmic and arhythmic items, where the *opti3* measure of melodic similarity was the dependent variable. By using statistical predictions from these models for all items in the item bank (i.e., including those that were not empirically tested), we were able to yield values which can represent difficulty, for each item.

### General discussion

Across two experiments, this paper described the development of an open-source infrastructure for testing sung recall. It employed ideas and approaches from both melodic memory (e.g., Sloboda & Parker, 1985) and singing accuracy (e.g., Pfordresher et al., 2015) perspectives, and hence, is able to facilitate research in both fields. The testing infrastructure builds upon existing routines for the measurement of musical abilities which are often limited to perceptual tests. Here, we offer a solution for extending research to musical production paradigms. This is extremely important to the future of musical testing: as recently conveyed by Okada and Slevc (2021), among others (Buren et al., 2021; Hallam & Creech, 2010; Silas & Müllensiefen, 2023), musical ability is not completely represented without testing musical production.

The work presented here provides a framework which allows a wide range of methods to score singing data with a battery of various measures, both new as well as previously described in the literature. The functionality enables researchers to create large item banks of melodic stimuli which are rich in features relevant to psychological processes, and sample from them in useful ways (e.g., to place them in the range of a singer whilst fulfilling other testing constraints). This is highly consequential for psychological testing since it allows researchers to connect relevant melodic features to task performance while maximizing heterogeneity and variance in the collected data, which is otherwise constrained by small item pools, which do not properly reflect the full variance in musical data. Our work also provides processes to maximize quality control, especially in online settings, which helps mitigate the occurrence of “dirty data” (Müllensiefen & Wiggins, 2011) in the first place. For instance, our analysis pipeline suggests that there is no difference between a user using an internal vs. an external microphone (see Appendix Fig. 5 and Table 15), which suggests our audio transcription is relatively robust, once certain constraints have been fulfilled (e.g., a certain SNR).

In the spirit of open-source software, this framework is openly available for use, and we encourage others to contribute to it. We emphasize the flexibility of the framework to be adapted in different settings, as has already been done (Gallant, 2022). In this way, the growing web documentation^35^ demonstrates how it is relatively easy to include new singing procedures (e.g., asking a participant to sing Happy Birthday) or add new scoring features into the analysis pipeline (i.e., taking the *pYIN* fundamental frequency and note onset information and score it via the *additional_scoring_measures* argument to the SAA test function), ultimately enabling researchers to test new hypotheses. There are also examples which show how the SAA can be included alongside other ability tests in a single timeline.

Beyond the methodological and theoretical contributions for music psychology, this research also has implications for the automatic assessment of musical performance (Abeßer et al., 2013, 2014; Dittmar et al., 2012; Knigge, 2010), which is becoming important in music education. In this context, tests of music production are designed to assess the musical production competences of students in schools, which can more objectively inform teachers of the ability level of students in their class, as well as provide the basis for specific teaching interventions. This is the eventual end goal of the current research agenda. We hope to support the wide interest in developing singing skills with the help of technology within academic settings, in a more open way than popular, but closed-source counterparts (e.g., 2022; “Smule,” 2022; “VoCo Vocal Coach on the App Store,” 2022). More broadly, we view adaptive testing as a step into tailored education via technology. Such education is not sufficient to serve the full range of musical experience, but it can certainly be very powerful when used as an educational tool.

**Fig. 2:**
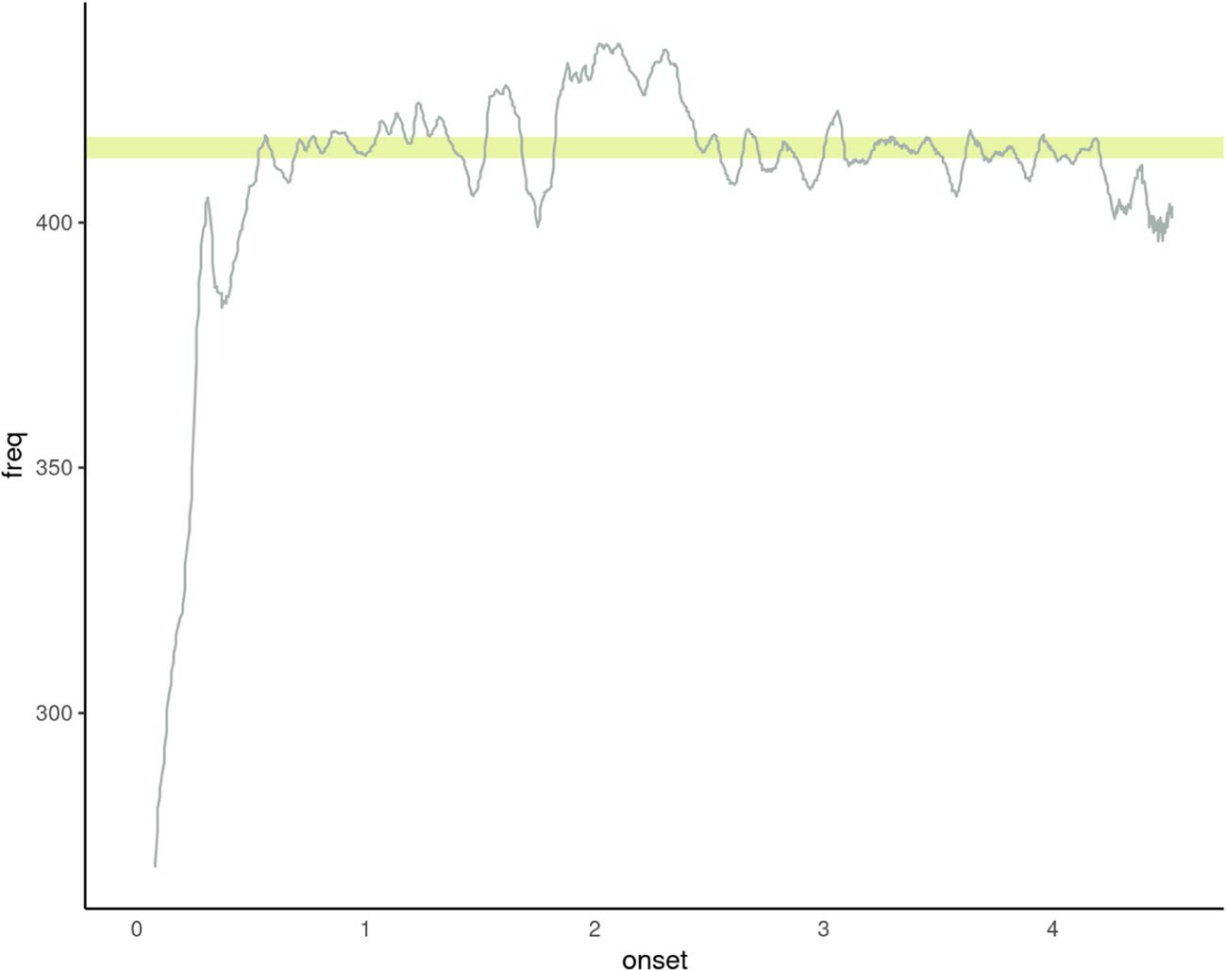
Examples of real data produced by the Singing Ability Assessment (SAA): long note singing feedback

**Fig. 3:**
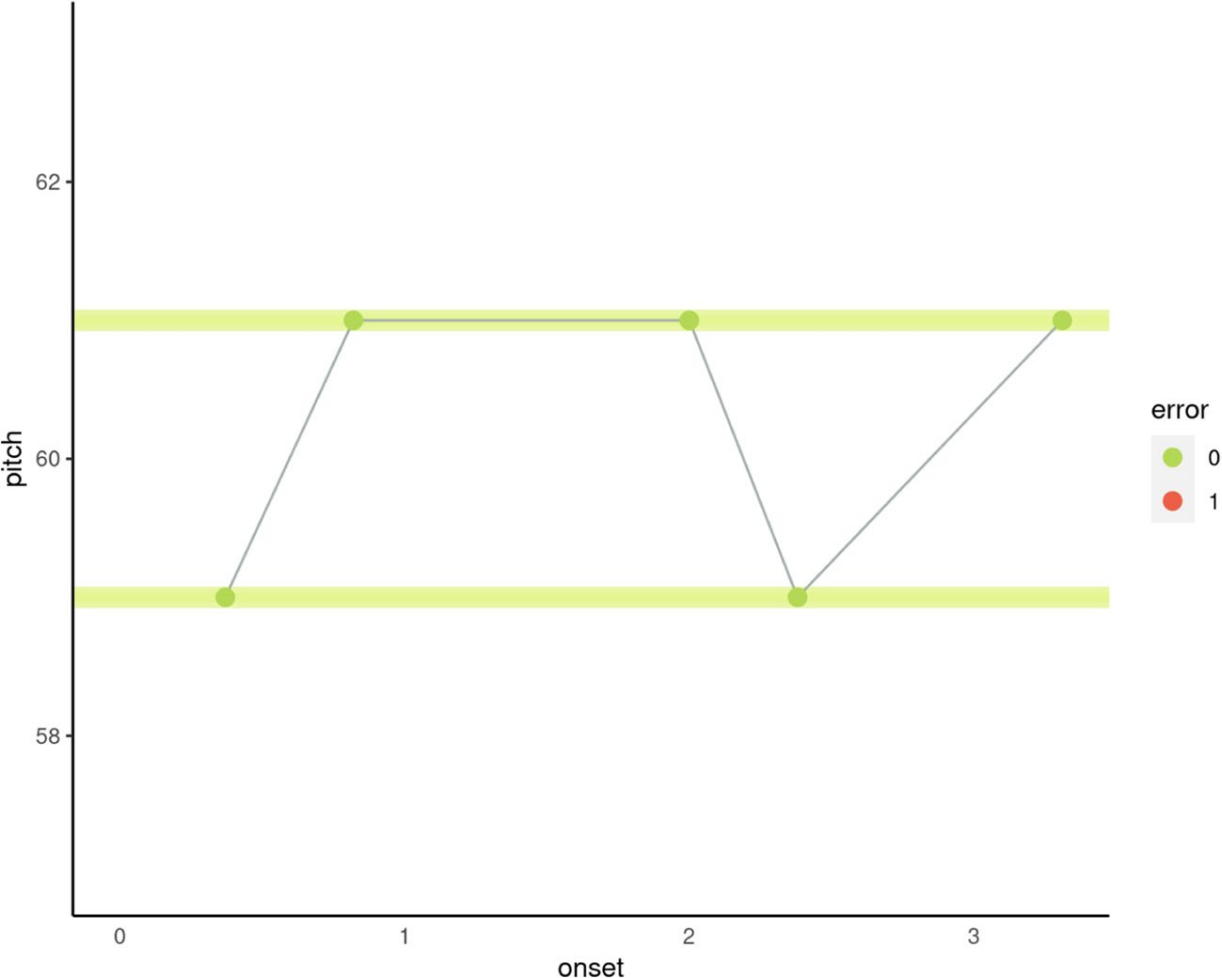
Examples of real data produced by the Singing Ability Assessment (SAA): Melodic singing feedback; *yellow-green lines* represent target pitches, *green points* correctly sung notes, *red points* incorrectly sung notes

**Fig. 4:**
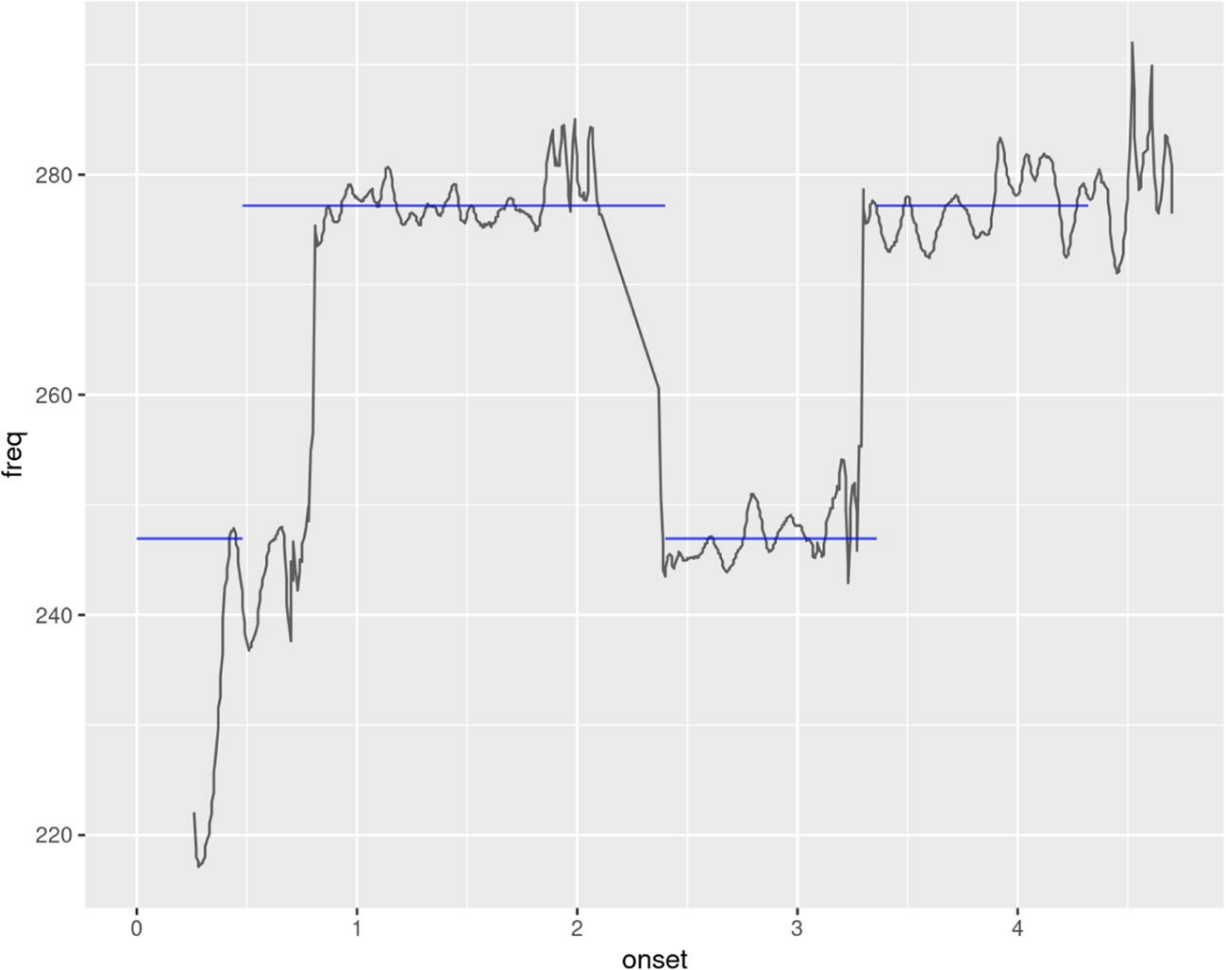
An alternative representation of melodic singing: the sung recall frequency curve in relation to target pitches (*solid rectangles*). In this case, the participant has sung a good approximation of the target pitches. Such a comparison of representations is the basis of the dynamic time warping distance measure we use. Note: the *solid rectangles* do not represent extracted onsets/pitches, but the target notes

The SAA is currently available for use here: https://saa.musicassessr.com. The code to produce this manuscript and analyses can be found here: https://github.com/sebsilas/SAA_Paper_2022. An online demo can be found here: https://adaptiveeartraining.com/SAA-demo. Table 11 lists the current features of the SAA. See Figs. 2, 3 and 4 for examples of feedback produced by the SAA.

**Table 11:** Summary of the Singing Ability Assessment’s features

Feature
Real-time fundamental frequency estimation and note onset detection
Real-time scoring with several different measures of singing ability
Triage participants based on their signal-to-noise at beginning of test
Collect a user’s vocal range and present stimuli to that range at test time
Use of multiple item banks
Item response theory-based modeling
Computerized adaptive testing
Optional performance feedback, including musical notation and audio feedback in the browser
Deploy easily alongside other ability tests
Suitable for online or in-person data collection
Scalable online server support via Amazon Web Services
Control test length (number of items) and constrain item features
Control melody sound (e.g., piano, tone, guitar)
Parameters to select different paradigms (e.g., arrhythmic, rhythmic) and number of attempts per melody
Relative ease to extend the battery with new scoring functions
Internalization (currently translated into German, Italian, Latvian, and Chinese in addition to English)

### Limitations

Our study has a number of limitations, some of which we will address in forthcoming research (we remind the reader the SAA is in continual development). First, our modeling approach is by no means the only way of relating melodic recall and singing accuracy variables to one another: there are many other possible frameworks and approaches. One of our main interests is in how other researchers will use the framework published here to further new modeling ideas. In particular, we are interested in employing structural equation modeling to more comprehensively relate our variable sets together. Second, our statistical modeling only applies to Western music. A future direction of this type of framework might be to extend it to different musical systems. We point readers to the other very innovative research in this regard (see Anglada-Tort et al., 2022; Jacoby et al., 2019). Third, we have not attempted to remove acoustic artifacts, or explored various audio manipulations before analysis. In one sense, this hands-off approach is a benefit: employing certain audio manipulation steps might introduce new artifacts in the process of removing others. However, we are keen to explore the audio cleaning steps taken in Anglada-Tort et al. (2022) with our data, to see how this may be able to improve our own analysis pipeline. We have already begun conducting such experiments, which we intend to present in a forthcoming paper, briefly suggested in the following section.

### Future Directions

In future work we aim to fully develop and implement an adaptive singing ability assessment (*aSAA*) test 36. This requires several new features and mechanisms, including the on-the-fly estimation of a participant’s singing ability and the enhancement of several components of the SAA. This may include the optimization of *opti3* as dependent variable to work better for singing data, and similarly, the parametric optimization of the *pYIN* fundamental frequency estimation algorithm for singing data. In addition, the participant’s ability to sing useable long notes could be tested more thoroughly at the beginning of the SAA test protocol in order to triage participants early in the test which will further maximize data quality and save participant’s time. Since, as documented commonly in performance research (Silm et al., 2020), and suggested in Experiment 2 of our paper, whereby not many participants optionally took more than one attempt at the same melody, any measures to improve participant effort will be valuable to the SAA. This is a main purpose of adaptive tests: shorter tests can maximize effort (e.g., with fewer trials, participants may be more likely to have more attempts at each trial). However, in parallel to the SAA development, we have also been exploring how we can make our tests more aesthetically engaging and maximize motivation (Silas, 2023), which we plan to extend to the SAA for future data collections. Finally, the new adaptive SAA will need to be validated and robust psychometric benchmarks will need to be derived from a large sample of participants of all singing abilities. Much in parallel, we are also developing the assessment procedures described above further in the context of data collected from people playing musical instruments, with the objective of eventually facilitating reliable real-time assessment and tailored music education (Silas et al., 2021).

## Figures and Tables

**Fig. 5:**
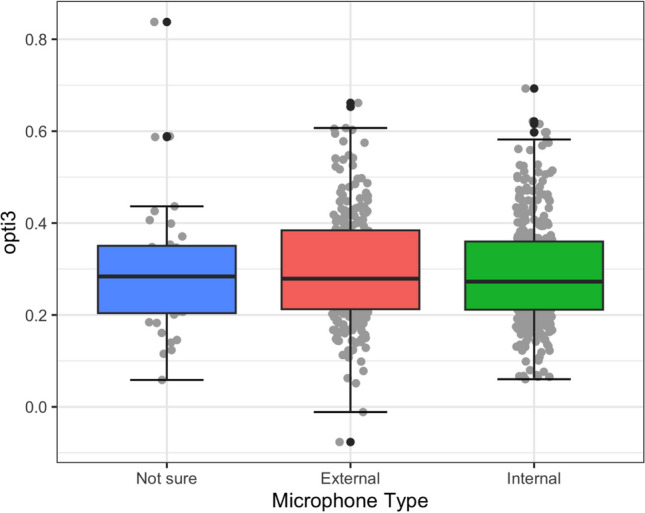
Boxplot, dot plot and SEM plot of opti3 for self-reported microphone type

## Data Availability

The code and data associated with this paper can be found at the following Github repositories: https://github.com/sebsilas/SAA_Paper_2022; https://github.com/sebsilas/SAA.

## Appendix 1

### Reliability of adaptive tests and the lengths that were used in Experiment 1, according to their original publications

Table 12

**Table 12:** Reliability of adaptive tests

Test	No. items	Reliability	Reliability type
JaJ	8	~ .78	Empirical
MDT	11	~ .62	Test–retest
PIAT	15	~ .60	Test–retest
PDCT	15	Not known	Not known
MPT	15	~ .60	Test–retest

Note that estimates are approximate due to being read from graphs visually

## Appendix 2

**Table 13:** Melodic features

Feature	Description	Equation	Reference
N	The length of the target melody.	-	-
log_freq	The log of the relative count of a frequency in the corpus	-	-
i.entropy	The average level of “information” or “surprise” in intervallic representations. Specifically, a variant of Shannon entropy on interval representations (Shannon, 1948)	$-\frac{\sum_{i}{f}_{i}\cdot {\mathrm{log}}_{2}{f}_{i}}{{\mathrm{log}}_{2}139}$	Müllensiefen, 2009
step.cont.loc.var	The mean absolute difference between adjacent values in the vector representing of step contour.	$\frac{\sum_{i=1}^{N-1}|{x}_{i+1}-{x}_{i}|}{N-1}$	Müllensiefen, 2009
d.entropy	The average level of “information” or “surprise” in rhythm values. Specifically, a variant of Shannon entropy on rhythmic representations (Shannon, 1948)	$-\frac{\sum_{i}{f}_{i}\cdot {\mathrm{log}}_{2}{f}_{i}}{{\mathrm{log}}_{2}140}$	Müllensiefen, 2009

*i.entropy* measures the average level of information or uncertainty of the distribution of pitch intervals in a melody. Large values of *i.entropy* are associated with melodies that use all pitch intervals a similar number of times over the course of the melody. Similarly, *d.entropy* measures the average level of information or uncertainty of the distribution of duration values of the notes in a melody. Melodies with large values of *d.entropy* will use many different note durations almost equally often. *step.cont.loc.var* is a measure of the variation of adjacent pitch values in a contour representation of a melody. Large values of *step.cont.loc.var* are associated with melodies that mainly use large intervals in their contour movements. *tonalness* measures how strongly a melody correlates with a single key center. It is derived by computing the correlation of a melody with all major and minor key centers and taking the largest correlation value from this set. A melody high on *tonalness* will clearly be in e.g., C major and have a large correlation value associated with that key

## Appendix 3

### SAA Dependent variables

Table 14

**Table 14:** Melodic dependent variables produced by the SAA

Measure	Definition
ngrukkon	Ukkonen measures for N-grams on raw pitch values
harmcore	Edit Distance of harmonic symbols per segment, obtained via Krumhansl’s tonality vectors.
rhythfuzz	Edit distance of classified length of melody tones.
opti3	3.027 * ngrukkon + 2.502 * rhythfuzz + 1.439 * harmcore
no_recalled_notes	The number of notes the participant produced in the trial.
no_correct_notes*	The number of correct notes a participant sang.
no_error_notes	The number of error notes a participant sang.
proportion_of_correct_note_events*	The proportion of recalled notes which were correct.
proportion_of_stimuli_notes_found*	The proportion of notes in the stimuli which were found.

^*^ this measure has an ‘octaves allowed’ partner, which compares pitch classes rather than MIDI pitches. Hence, it does not matter what octave something is sung in. Variable names use snake case, corresponding to their naming inside the SAA software.

The *ngrukkon* similarity measure measures the difference in the occurrence of short pitch sequences between the two melodies to be compared. It is computed in several steps. First, the occurrence frequency of N-grams (i.e., sequences of three notes) is tallied for each of both melodies. Subsequently, the difference between the occurrence of the same N-grams in melody A and melody B is computed and these occurrence differences are summed up. Finally, the resulting value is normalized by the maximum number of possible N-grams and subtracted from 1 to yield a similarity measure ranging from 0 to 1. Harmonic similarity is measured by the *harmcore* measure. This measure is based on the chords implied by a melodic sequence, taking pitches and durations into account. Implied harmonies are computed using the Krumhansl–Schmuckler algorithm (Krumhansl, 1990) and the harmonic sequences of the two melodies are compared by computing the number of operations necessary to transform one harmonic sequence into the other sequence via the edit distance. Finally, likewise, rhythmic similarity is computed by first categorizing the durations of the notes of both melodies (known as “fuzzification”) and then applying the edit distance to measure the distance between the two sequences of categorized durations. The resulting measure of rhythmic similarity is called *rhythfuzz* (Müllensiefen & Frieler, 2004b). Note that *rhythfuzz* does not take metric information into account and works solely on the basis of (relative) note durations. Similarly, *ngrukkon* works with interval information and is hence invariant to transposition.

Based on the perceptual data collected by Müllensiefen and Frieler (2004b), the three individual measures are weighted and combined to form a single aggregate measure of melodic similarity, *opti3*. Hence, *opti3* is sensitive to similarities and differences in three important aspects of melodic perception (pitch intervals, harmony, rhythm). We note that all three individual measures (*ngrukkon*, *harmcore*, *rhythfuzz*) can take values between 0 (= no similarity) and 1 (= identity) and are length-normalized by considering the number of elements of the longer melody. *opti3* then comprises (Müllensiefen & Frieler, 2004b):

3 $$opti3=0.505\cdot \mathtt{n}\mathtt{g}\mathtt{r}\mathtt{u}\mathtt{k}\mathtt{k}\mathtt{o}\mathtt{n}+0.417\cdot \mathtt{r}\mathtt{h}\mathtt{y}\mathtt{t}\mathtt{h}\mathtt{f}\mathtt{u}\mathtt{z}\mathtt{z}+0.24\cdot \mathtt{h}\mathtt{a}\mathtt{r}\mathtt{m}\mathtt{c}\mathtt{o}\mathtt{r}\mathtt{e}-0.146$$

Note that we here present the normalized weights, which constrain the values to be [0,1].

## Appendix 4

### Comparison of internal vs. external microphone selection

When comparing the effect of using an internal vs. external microphone, as self-selected by the user at the beginning of entry to the SAA, our results suggest there is no difference in *opti3* scores.

A mixed-effects model with microphone type as fixed-effects categorical predictor (External, Internal, Not sure) and participant as a random effect intercept revealed that there was no statistically significant difference in *opti3* scores between the microphone types (*p* < .05). See the table below for model parameters.

**Table 15:** Mixed-effects model with *opti3* as dependent variable, microphone type as fixed-effects categorical predictor (External, Internal, Not sure) and participant as random effects intercept

Term	$\widehat{\beta }$	95% CI	$t$	$df$	$p$
Intercept	0.30	[0.29, 0.32]	45.68	770.33	< .001***
Microphone typeInternal	– 0.01	[– 0.03, 0.01]	– 1.32	973.95	.186
Microphone typeNot sure	– 0.01	[– 0.04, 0.03]	– 0.46	1,534.15	.643

The average *opti3* score for external microphones (M = 0.31, SD = 0.24) had a fractionally higher *opti3* score than internal microphones (M = 0.30, SD = 0.23). This suggests that the combination of *pYIN* and *opti3* in our analysis pipeline are robust to the important feature of microphone type in a user’s setup

*p* < .001***

## Footnotes

1 Note that the *Singing Ability Assessment* test presented in this paper has already been adapted to work in the context of the *AIRS* battery (Gallant, 2022)

2 At the time of writing, our test has been translated into German, Italian, Latvian, and Chinese, in addition to English.

3 These can be created with the *itembankr R* package from *.mid* files, *.musicxml* files or a dataframe of melody pitches and frequencies. See: https://github.com/sebsilas/itembankr.

4 https://saa.musicassessr.com/.

5 https://github.com/sebsilas/pyin.

6 https://sebsilas.github.io/musicassessr/.

7 The scoring functionality can work both in a real-time test as well as in batch-mode after audio recordings have been made.

8 https://github.com/sebsilas/itembankr.

9 https://github.com/sebsilas/Berkowitz

10 https://saa.musicassessr.com.

11 See https://shiny.gold-msi.org/longgold_demo/ and https://testing.musikpsychologie.de/dots_home/.

12 See https://github.com/sebsilas/PDT.

13 https://www.slicethepie.com/.

14 https://aws.amazon.com/ec2/.

15 https://www.rstudio.com/products/shiny/shiny-server/.

16 https://aws.amazon.com/s3/.

17 Note that the S3 bucket approach was eventually discontinued.

18 An N-gram is a contiguous subset of a sequence (e.g., “ABIL” is an N-gram of “SINGINGABILITY”)

19 Note that subjective self-reports of singing ability are a different kind of measure of singing ability to the objective measurement and computational approach taken in this paper. Yet, it is a clear target to obtain construct validity.

20 “Complex” refers to the nature of the tone, which contains harmonics, rather than being a “simple” sine tone, which contains no harmonics.

21 Accessible here: https://github.com/sebsilas/PDT.

22 It is possible to use the protocol to test memory over longer timespans too.

23 Later updates allowed us to record directly to .wav files.

24 An interval is the musical distance between notes in terms of pitch.

25 See Müllensiefen (2009) for formal definitions.

26 With respect to the Berkowitz corpus from which it was derived.

27 Note that snake case variable names are presented in accordance with their naming in our *R* packages, for easy reference.

28 For the implementation of these scoring methods, see https://github.com/sebsilas/musicassessr/blob/master/R/scoring_simile.R.

29 http://saa.musicassessr.com/reference/SAA.html.

30 Whether to use an SNR test is controlled via the *SNR_test* argument to *SAA* functions; whether to allow multiple attempts, or disqualify on the first failure is controlled by the *allow_repeat_SNR_tests* argument; whether to display the captured SNR as feedback to the participant is controlled via the *report_SNR* argument.

31 This can be altered via the min_SNR argument to the *SAA* test function.

32 The *adjust_range* argument allows this to be “corrected” if, via some heuristics, it seems that the participant did not complete the task appropriately (e.g., sings a “high” note lower than a “low” note).

33 https://github.com/sebsilas/Berkowitz.

34 Note: negative coefficients appear when the measure appears to represent deviation from a target i.e., higher score = more error.

35 https://saa.musicassessr.com/articles.

36 However, readers can already view and explore a prototype here: https://adaptiveeartraining.com/aSAA.
